# The Effect of Chronic Mild Stress and Venlafaxine on the Expression and Methylation Levels of Genes Involved in the Tryptophan Catabolites Pathway in the Blood and Brain Structures of Rats

**DOI:** 10.1007/s12031-020-01563-2

**Published:** 2020-05-13

**Authors:** Paulina Wigner, Ewelina Synowiec, Paweł Jóźwiak, Piotr Czarny, Michał Bijak, Katarzyna Białek, Janusz Szemraj, Piotr Gruca, Mariusz Papp, Tomasz Śliwiński

**Affiliations:** 1 Faculty of Biology and Environmental Protection, Laboratory of Medical Genetics, University of Lodz, Pomorska 141/143, 90-236 Lodz, Poland; 2 Faculty of Biology and Environmental Protection, Department of Cytobiochemistry, University of Lodz, Lodz, Poland; 3 Department of Medical Biochemistry, Medical University of Lodz, Lodz, Poland; 4 Faculty of Biology and Environmental Protection, Department of General Biochemistry, University of Lodz, Lodz, Poland; 5 Polish Academy of Sciences, Institute of Pharmacology, Krakow, Poland

**Keywords:** Chronic mild stress model of depression, Venlafaxine, Tryptophan catabolites pathway, Gene expression and methylation

## Abstract

A growing body of evidence suggests that depression may be associated with impairment of the tryptophan catabolites (TRYCATs) pathway. The present study investigated the effects of the chronic administration of venlafaxine on the expression and methylation status of *Katl*, *Tph1/2*, *Ido1*, *Kmo* and *Kynu* in the brain and blood of rats exposed to the CMS model of depression. The rats were subjected to the CMS procedure for 2 or 7 weeks and administered venlafaxine (10 mg/kg/day, IP) for 5 weeks. mRNA and protein expression and the methylation status of gene promoters in PBMCs and six brain structures were evaluated and analysed using the TaqMan Gene Expression Assay and Western blotting, and methylation-sensitive high-resolution melting (MS-HRM), respectively. We found that the CMS procedure increased *KatI* expression in the midbrain and *KatII* expression in the midbrain and the amygdala, while venlafaxine administration decreased *KatII* expression in the hypothalamus and the cerebral cortex. The methylation status of the *Tph1* and *Kmo* promoters in peripheral blood mononuclear cells (PBMCs) was significantly increased in the stressed group after antidepressant therapy. The protein levels of Tph1 and Ido1 were decreased following venlafaxine administration. Our results confirmed that CMS and venlafaxine modulate the expression levels and methylation status of genes involved in the TRYCATs pathway.

**Electronic supplementary material:**

The online version of this article (10.1007/s12031-020-01563-2) contains supplementary material, which is available to authorized users.

## Introduction

According to a World Health Organization (WHO) report, 350 million people globally suffer from depression, and 800,000 people commit suicide every year (James et al. 2018; Wang et al. 2007). Moreover, it is estimated that by 2020, depression will be the main cause of significant health, economic and social burdens (Murray and Lopez 1997). Unfortunately, despite extensive studies, the pathogenesis of depression is not fully known. A growing body of evidence has suggested that the mechanisms of this disease are associated with disorders of the tryptophan catabolites (TRYCATs) pathway (Maes et al. 2011a, b). A detailed description of the TRYCATs pathway is presented in previous studies have confirmed that depression is associated with reduced levels of tryptophan and neuroprotective kynurenic acid and elevated concentrations of neurotoxic metabolites, including 3-hydroxykynurenine, quinolinic acid and anthranilic acid, as well as with disorders of enzymes involved in tryptophan metabolism, including increased activity of indoleamine 2,3-dioxygenase 1 (IDO 1) and tryptophan 2,3-dioxygenase 2 (TDO 2), and decreased activity of kynurenine aminotransferase 1 and 2 (KATI and KATII) (Maes et al. 2011a; Ogawa et al. 2014; Kwidzinski and Bechmann 2007). IDO 1 and TDO 2 are rate-limiting enzymes which catalyse the oxidation of l-tryptophan to *N*-formylkynurenine. TDO 2 is expressed in the liver, whereas IDO 1 is expressed in the placenta, lungs, brain and blood (Hayaishi 1976; Watanabe et al. 1980). Kynurenic acid is a product of the reaction catalysed by KATI or KATII from kynurenine. The next important enzymes associated with depression are tryptophan hydroxylase 1 and 2 (TPH1, TPH2). TPH is involved in the initial and rate-limiting step in the synthesis of serotonin and melatonin. The enzyme catalyses the monooxygenation of tryptophan to 5-hydroxytryptophan (Wigner et al. 2018a). In turn, kynurenine-3-monooxygenase (KMO) catalyses the hydroxylation of l-kynurenine to neurotoxic 3-hydroxykynurenine (Breton et al. 2000). In previous studies, polymorphisms in the genes that encode TPH1, TPH2, IDO1 and KMO have been found to modulate the risk of depression development (Wigner et al. 2018a, b; Lezheiko et al. 2016). Thus, studying TRYCATs pathway in depression can provide new diagnostic biomarkers of this disease and may allow for the development of promising new personalized antidepressive drugs in the future (Smith 2013). This is of particular importance, as antidepressant treatment is not effective in approximately 30% of depressed patients (Joffe et al. 1996), and recent studies have shown that a critical drop in the level of tryptophan is associated with the development of drug-resistant depression (Smith 2013). Interestingly, the occurrence of *TPH1*, *TPH2* and *KATI* polymorphisms may be associated with a lack of response to traditional therapy based on the application of selective serotonin reuptake inhibitors (SSRIs). The c.-173A>T (rs10488682) polymorphism is localized in the promoter region of *TPH1* and may decrease the activity of the promoter, affecting the transcription level of *TPH1*. Moreover, the 844G>T (rs4576025) *TPH2* polymorphism may alter DNA–protein interactions and may affect the transcription level. The presence of the T allele is associated with reduced *TPH2* promoter activity (Smith 2013; Zhang et al. 2005; Wigner et al. 2018a, b).

In addition to SSRIs, serotonin-norepinephrine reuptake inhibitors (SNRIs), including venlafaxine, are the first line of depression therapy. Venlafaxine is approved by the US Food and Drug Administration (FDA) to treat and manage symptoms of depression, general anxiety disorder, social phobia and panic disorder (Safarova et al. 2018). Venlafaxine is a bicyclic phenylethylamine compound and works by blocking the transporter “reuptake” proteins for key neurotransmitters, including serotonin and norepinephrine, thereby leaving more active neurotransmitters in the synapse. Moreover, venlafaxine is a more potent inhibitor of serotonin reuptake than norepinephrine reuptake (Saad et al. 2019). Additionally, methylation status and mRNA expression level may be modulated by stress and antidepressant therapy. Previous studies showed that the expression and activity of DNA methyltransferases were increased in patients with depression. In the case of animals exposed to stress stimuli, studies confirmed that the level of DNA methyltransferases was increased in the prefrontal cortex and hippocampus (Nagy et al. 2018; Webb et al. 2020). The effectiveness of antidepressants may also be associated with differential methylation of the CYP450 enzymes of the liver which metabolize antidepressant drugs. Elevated methylation status of the promoter region was found to correlate with low or no transcription. Methylation levels may also differ between different tissue types and between normal cells and diseased cells from the same tissue (Suzuki and Bird 2008; Habano et al. 2015; Tili et al. 2015). Thus, this study aimed to investigate whether (i) the chronic mild stress (CMS) procedure changes the expression of genes involved in the TRYCATs pathway at the mRNA and protein levels and causes epigenetic changes, i.e. methylation level of these gene promoters in peripheral blood mononuclear cells (PBMCs) and in selected brain structures (hippocampus, amygdala, midbrain, hypothalamus, cerebral cortex and basal ganglia); and (ii) chronic administration of the serotonin-norepinephrine reuptake inhibitor venlafaxine affects the expression and methylation status of these genes. The latter point has important clinical implications, since there is a great need for peripheral markers that would enable earlier diagnosis, more precise prognosis of pharmacotherapy outcome, and more personalized therapies for mood disorders. All of the genes analysed in our study are located on chromosomes significantly associated with depression (Supplementary Table 1). Buczko et al. (2005) presented the correct course of the TRYCATs.

## Materials and Methods

### Animals

Male Wistar Han rats were obtained from Charles River (Germany). The animals were singly housed with free access to food and water and kept on a 12-h light/dark cycle (lights on at 8:00) at a controlled temperature (22 ± 2.0 °C) and humidity (50 ± 5%). All procedures and tests used in this study were approved by the Bioethical Committee of the Institute of Pharmacology of the Polish Academy of Sciences in Krakow (Poland) and were conducted in compliance with the rules and principles of the 86/609/EEC directive.

### Chronic Mild Stress Procedure

CMS experiments were performed according to the method described previously (Papp 2012). Briefly, the animals (approximately 220 g at the start of the procedure) were first trained to consume 1% sucrose solution in 7 weekly baseline tests, in which they received the solution for 1 h following 14 h of food and water deprivation. The animals were then divided into two matched groups. The first group included animals subjected to the stress procedure for 2 or 7 weeks. Each week of the stress procedure involved two periods of food or water deprivation, two periods of 45-degree cage tilt, two periods of intermittent illumination (lights on and off every 2 h), two periods of soiled cage (250 ml water in sawdust bedding), one period of paired housing, two periods of low-intensity stroboscopic illumination (150 flashes/min), and three periods of no stress. The animals from the control group had free access to food and water, except for the period of food and water deprivation before the sucrose consumption tests. They were kept in a separate room and had no contact with the stressed rats. After the initial 2 weeks of stress, i.e., when the decrease in sucrose intake stabilized, the animals were either decapitated or further divided into subgroups and administered vehicle (1 ml/kg, IP) or venlafaxine (10 mg/kg, IP) daily for 5 weeks. Finally, the animals were decapitated 24 h after the last sucrose test, and samples of blood and brain structures were collected.

### Drugs

Venlafaxine HCl (Carbosynth Ltd., Compton, Berkshire, UK) was dissolved in 0.9% sterile saline, which was used for vehicle injection, and was administered intraperitoneally (IP) in a volume of 1 ml/kg of body weight at a dose of 10 mg/kg, as used previously (Papp et al. 2017; Papp et al. 2019).

### Specimen Collection

After decapitation, blood samples were collected in 5-ml Vacutainer tubes containing EDTA and stored at −20 °C. Next, differential migration of cells during centrifugation with Gradisol L (Aqua-Med, Lodz, Poland) was applied to isolate peripheral blood mononuclear cells (PBMCs). Finally, after centrifugation (400×*g*, 30 min, 4 °C), the PBMC pellet was stored at −20 °C until further analysis. The hippocampus, amygdala, midbrain, hypothalamus, cerebral cortex and basal ganglia were also isolated, rapidly frozen in liquid nitrogen and stored at −80 °C until further analysis. A FastGene^®^ tissue grinder (Nippon Genetics Europe, Düren, Germany) was then used to homogenize the tissues and prepare DNA, RNA and protein specimens.

### RNA Isolation, cDNA Synthesis and mRNA Expression Levels

Commercial spin column methods (GenElute Mammalian Total RNA Miniprep Kit, Sigma-Aldrich, and ISOLATE II RNA/DNA/Protein Kit, Bioline) were used to isolate RNA from the PBMCs and frozen brain structures in accordance with the manufacturers’ instructions. The total concentration and quality of the RNA samples were determined by comparing the absorbance values at 260 nm and 280 nm, after which the samples were stored at −20 °C until use. The next step involved the synthesis of cDNA products from total RNA using an Applied Biosystems High-Capacity cDNA Reverse Transcription Kit (Foster City, CA, USA) according to the manufacturer’s instructions. Briefly, the reverse transcription reaction consisted of MultiScribe^®^ Reverse Transcriptase, 10× RT random primers, 25× dNTP Mix (100 mM), nuclease-free water, 10× RT buffer and total RNA (0.5 ng/μl). The conditions of the cDNA synthesis reaction executed in a C1000™ programmed thermal cycler (Bio-Rad Laboratories, Inc., Hercules, CA, USA) were as follows: 10 min at 25 °C (enzyme activation), 37 °C for 120 min (proper synthesis of cDNA), and 85 °C for 5 min (enzyme inactivation). The level of mRNA expression was measured by quantitative reverse transcription polymerase chain reaction (RT-qPCR) using commercially available TaqMan Universal Master Mix, no UNG, and species-specific TaqMan Gene Expression Assay (Thermo Fisher Scientific, Waltham, MA, USA). The target genes included *KatI* (assay ID Rn01439192_m1), *KatII* (assay ID Rn00567882_m1), *Tph1* (assay ID Rn00598017_m1), *Tph2* (assay ID Rn01476867_m1), *Ido1* (assay ID Rn01482210_m1), *Kmo* (assay ID Rn01411937_m1) and *Kynu* (assay ID Rn01449532_m1), As an internal mRNA control, we used 18S ribosomal RNA (18S, Applied Biosystems, CA, USA), and the mRNA expression of 18S ribosomal RNA was used to normalize the target gene expression levels. A quantitative RT-PCR reaction was carried out using a CFX96™ Real-Time PCR Detection System Thermal Cycler (Bio-Rad Laboratories, Inc., Hercules, CA, USA). The two-step amplification conditions were as follows: 10 min at 95 °C followed by 60 cycles at 95 °C for 30 s and 1 min at 60 °C. The experiments were performed in duplicate for each sample. Gene expression was calculated in relation to that of the reference gene (ΔC_t sample_ = C_t target gene_ − C_t reference gene_). Next, the levels of gene expression were given as a the ratio calculated as fold = 2^-ΔCt sample^. The fold change in expression caused by venlafaxine administration was calculated using the 2^-ΔΔCt^ method (Schmittgen and Livak 2008).

### DNA Isolation and Methylation and HRM Analysis

Genomic DNA was isolated from the PBMCs and brain structures according to the manufacturer’s instructions supplied with the QIAamp DNA Mini Kit (Qiagen, Hilden, Germany) and ISOLATE II RNA/DNA/Protein Kit (Bioline, Alvinston, Canada), and stored at −20 °C until use. The quantity and quality of the isolated DNA samples were measured by a spectrophotometer. Methylation-sensitive high-resolution melting (MS-HRM) was used to assess the methylation level of the gene promoter region (Wojdacz and Dobrovic 2007; Wojdacz et al. 2008). Therefore, the EMBOSS Cpgplot bioinformatics tool (https://www.ebi.ac.uk/Tools/seqstats/emboss_cpgplot/, Settings: Window: 100, Shift: 1, Obs./Exp.: 0.6, GC content: 50%) was used to predict CpG islands in the promoter regions of all the studied genes. The next step involved designing primers in MethPrimer 2 (http://www.urogene.org/methprimer2/) according to the recommendations provided by Wojdacz et al. (2009). Supplementary Table 2 provides the specifications of the designed primers. Bisulfite conversion was then performed using the CiTi Converter DNA Methylation Kit (A&A Biotechnology, Gdynia, Poland) according to the manufacturer’s protocols. Real-time PCR amplification was carried out on the Bio-Rad CFX96 Real-Time PCR Detection System (Bio-Rad Laboratories, Inc., Hercules, CA, USA) with the following thermal cycling conditions: initial activation for 12 min at 95 °C, 45 cycles at 95 °C for 15 s; annealing at the optimal primer temperature (tested experimentally) for 20 s (see Supplementary Table 1 for the characteristics of the primers), and elongation at 72 °C for 20 s. The HRM analysis involved denaturation at 95 °C for 15 s, re-annealing at 60 °C for 1 min, and melting from 60 to 95 °C at a ramp rate of 0.2 °C every 2 s. Each PCR was composed of 5x HOT FIREPol^®^ EvaGreen^®^ HRM Mix (no ROX) (Solis BioDyne, Tartu, Estonia), 500 nM of each primer and 10 ng of DNA after bisulfite modification (theoretical calculation). Finally, Bio-Rad Precision Melt Analysis Software was used to analyse the obtained data. In addition, unmethylated and methylated bisulfite-converted control DNA (CpGenome™ Rat Methylated Genomic DNA Standard, Merck Millipore, Burlington, MA, USA, and CpGenome™ Rat Unmethylated Genomic DNA Standard, Merck Millipore) was used in different ratios for HRM calibration (0%, 10%, 25%, 50%, 75% and 100% methylated controls).

### Western Blot Analysis

Protein expression levels in the structures of rat brain tissues were estimated using Western blot analysis as described previously (Laemmli 1970). A FastGene^®^ tissue grinder (Nippon Genetics Europe, Düren, Germany) was used for the homogenization of frozen brain samples in RIPA buffer (10 mM Tris-HCl, pH 8.0, 1 mM EDTA, 1% Triton X-100, 0.1% sodium deoxycholate, 0.1% SDS, and 10 Mm NaCl) containing 1 mM phenylmethylsulfonyl fluoride (PMSF, serine protease inhibitor). Following double sonication and centrifugation (5000 rpm, 5 min, 4 °C), the supernatant containing the protein was collected. The protein concentration was measured based on the modified Lowry procedure using bovine serum albumin (BSA) as a standard (Lowry et al. 1951). Samples of brain homogenates (50 μg/lane) were resolved by 10% SDS-PAGE and then electroblotted onto Immobilon-P membranes (Millipore, Bedford, MA, USA) as previously described (Towbin et al. 1979). After blocking at room temperature for 1 h and washing three times with TBST (Tris-buffered saline with Tween-20), the blots were incubated overnight at 4 °C with primary antibodies (incubation for 2 h at room temperature except for the anti-β-actin antibody) diluted according to the manufacturer’s protocol. The antibody specifications are presented in Supplementary Table 3. After incubation, the blots were washed three times with TBST and then incubated for 1 h with appropriate secondary antibodies conjugated to horseradish peroxidase (1:6000 dilution). Finally, the membranes were again washed with TBST and incubated with peroxidase substrate solution (Thermo Fisher Scientific, Waltham, MA, USA). The proteins were visualized on X-ray film by enhanced chemiluminescence. Densitometry analysis of protein bands was performed with Gel-Pro^®^ Analyzer Software (Media Cybernetics, Inc., Rockville, MD, USA). The protein expression levels were normalized to the reference protein, i.e., beta-actin (ACTB; IOD_gene/_IOD_ACTB_).

### Statistical Analysis

All data are presented as the means ± standard error of means. The normality of the data was confirmed using the Shapiro–Wilk test. Differences between samples with normal distribution were verified using analysis of variance (ANOVA), whereas the Kruskal–Wallis test combined with a multiple comparison of average ranks was used to determine differences between samples with non-normal distribution. Subsequently, the Tukey test was used as the post hoc test. In the case of differences in mRNA expression and methylation levels between the blood and brain, a *t* test was used. *P* values <0.05 were considered statistically significant. The results were analysed using Statistica 12 (StatSoft, Tulsa, OK, USA), SigmaPlot 11.0 (Systat Software Inc., San Jose, CA, USA) and GraphPad Prism 5.0 (GraphPad Software, Inc., La Jolla, CA, USA).

## Results

### The Effect of CMS and Venlafaxine on Sucrose Intake

**Fig. 1:**
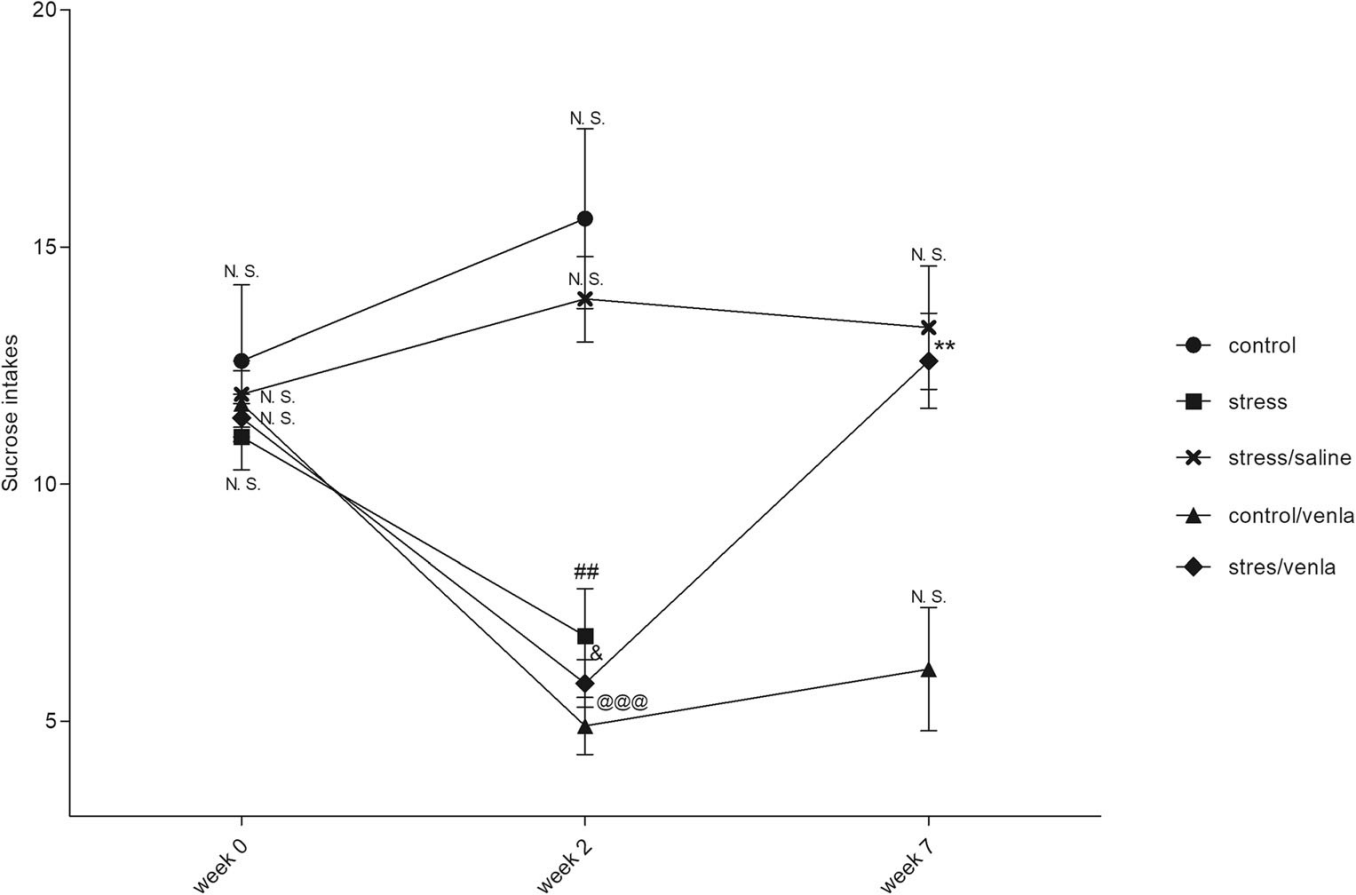
Sucrose intake in animals exposed to CMS for 2 weeks (week 2) and in animals exposed to CMS for 7 weeks (week 7) and administered vehicle (1 ml/kg) or venlafaxine (10 mg/kg) for 5 weeks. The data represent means ± SEM. *N* = 6. ^##^*p* < 0.01; relative to week 2 in the stressed group. ^&^*p* < 0.05; relative to week 2 in the stressed/venla group. **p < 0.01; relative to week 2 in the stressed/venla group. ^@@@^*p* < 0.001; relative to week 0 in the control/venla group. *N.S.* no significant differences between studied groups

Before the stress was initiated (week 0), the consumption of 1% sucrose solution was comparable in all groups. As shown in Fig. 1, after 2 weeks of initial stress, the rats showed an approximately 40% decrease in sucrose intake (*p* < 0.01), whereas in vehicle-treated stressed animals, sucrose intake remained at a similar level until the end of the experiment (week 7). The chronic administration of venlafaxine (10 mg/kg, IP) normalized the decreased intake in the stressed rats (*p* < 0.01) and had no effect on the behaviour of non-stressed control animals. Neither the stress procedure nor venlafaxine treatment had a significant effect on the body weight of the animals from any of the studied groups (data not shown).

### mRNA Expression

#### Gene Expression in Brain Structures and PBMCs

**Fig. 2:**
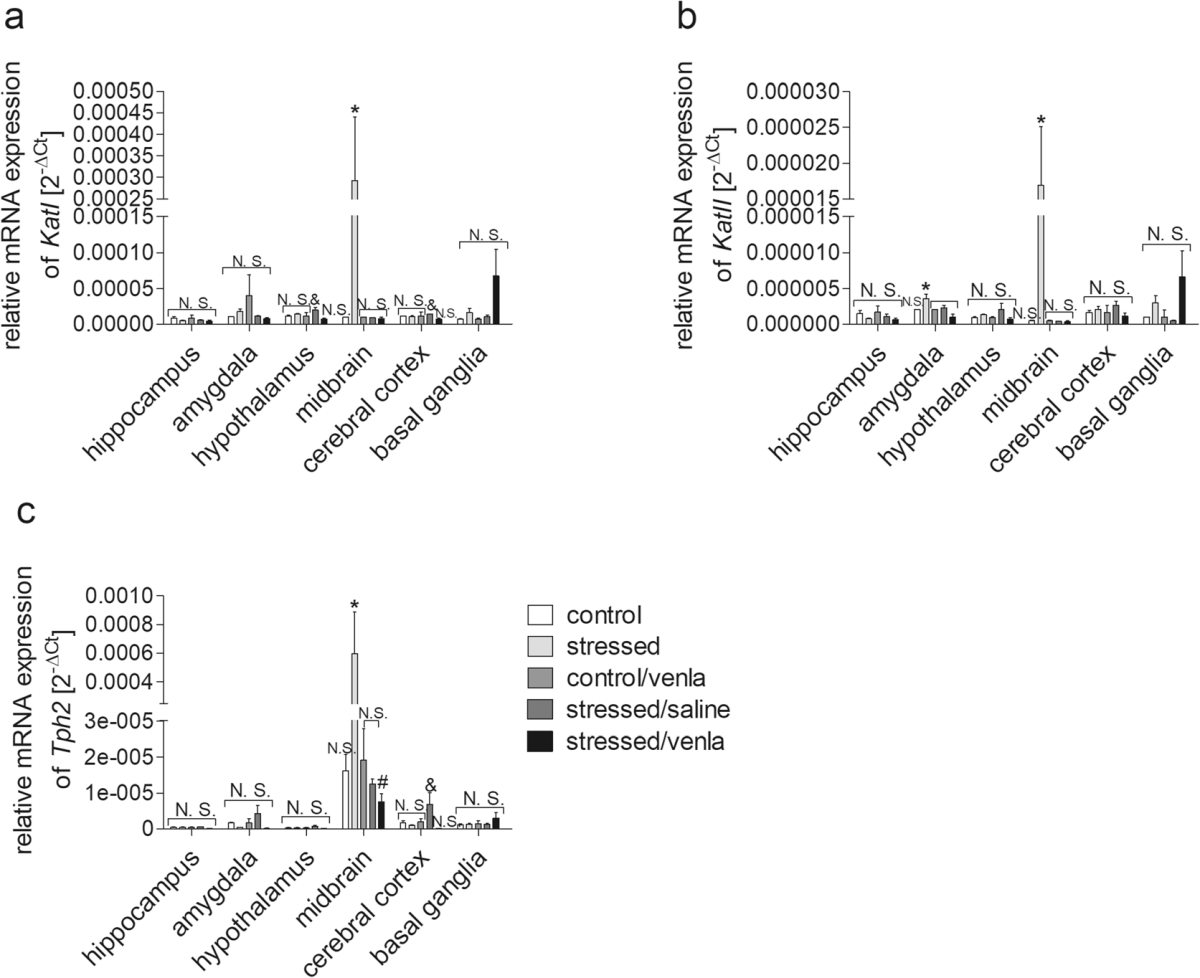
mRNA expression of *KatI* (**a**), *KatII* (**b**) and *Tph2* (**c**) genes in brain structures of animals exposed to CMS for 2 weeks (control, stressed) and in animals exposed to CMS for 7 weeks and administered vehicle (1 ml/kg) or venlafaxine (10 mg/kg) for 5 weeks (control/venla, stressed/saline, stressed/venla). The expression of either gene was normalized to the 18S gene, and relative gene expression levels were estimated using a 2^-ΔCt (Ct^_gene_^–Ct^_18S_^)^ method. N = 6. ^#^*p* < 0.05 for differences between stressed and stressed/venla group, *p < 0.05 stressed and control groups, ^&^*p* < 0.05 for differences between stressed/saline and stressed/venla groups, and ^#^*p* for differences between controls and stressed groups. *N.S.* no significant differences between studied groups

As shown in Fig. 2 and Supplementary Fig. 1, the effect of the CMS procedure and venlafaxine treatment on *Tph1*, *Tph2*, *KatI*, *KatII*, *Ido1*, *Kmo* and *Kynu* mRNA expression was dependent on the tissue and brain structure. The CMS procedure increased *KatI* expression in the midbrain (*p* < 0.05), while venlafaxine decreased it in the hypothalamus (*p* < 0.05) and the cerebral cortex (*p* < 0.05) in stressed rats. The amygdala (*p* < 0.05) and midbrain (*p* < 0.05) of the stressed rats exhibited elevated *KatII* expression. mRNA expression of the *Tph2* gene was elevated in the midbrain of the stressed group as compared with controls, while reduced expression was observed following administration of venlafaxine in stressed rats (*p* < 0.05). *Tph2* expression in the cerebral cortex was lower after venlafaxine administration than after saline treatment (*p* < 0.05). On the other hand, neither stress nor venlafaxine had a significant effect on the expression of any of the studied genes in PBMCs (Supplementary Fig. 2).

#### The Effect of Venlafaxine on Gene Expression in PBMCs and Brain Structures

As shown in Supplementary Fig. 3, venlafaxine caused an increase in *KatII* and *Tph2* expression in the hippocampus amygdala, hypothalamus, midbrain and cerebral cortex (*p* < 0.001) and a decrease in *Kmo* expression in the hypothalamus of stressed rats (*p* < 0.01).

### Methylation of the Studied Gene Promoters

#### Methylation in PBMCs and Brain Structures

**Fig. 3:**
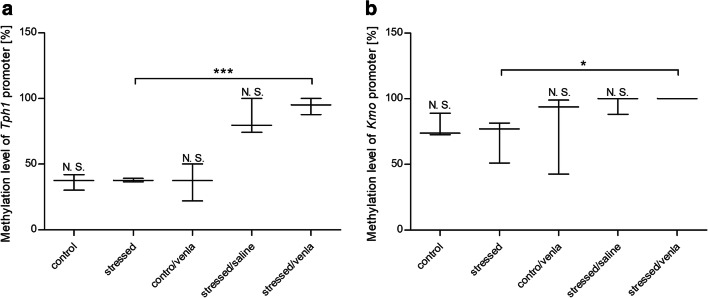
Methylation level of *Tph1* promoter (**a**) and *Kmo* promoter (**b**) in PBMCs of animals exposed to CMS for 2 weeks (control, stressed) and in animals exposed to CMS for 7 weeks and treated with vehicle (1 ml/kg) or venlafaxine (10 mg/kg) for 5 weeks (control/venla, stressed/saline, stressed/venla). Data represent means ± SEM. *N* = 6. *p < 0.05 and ***p < 0.001 for differences between stressed and stressed/venla groups. *N.S.* no significant differences between studied groups

**Fig. 4:**
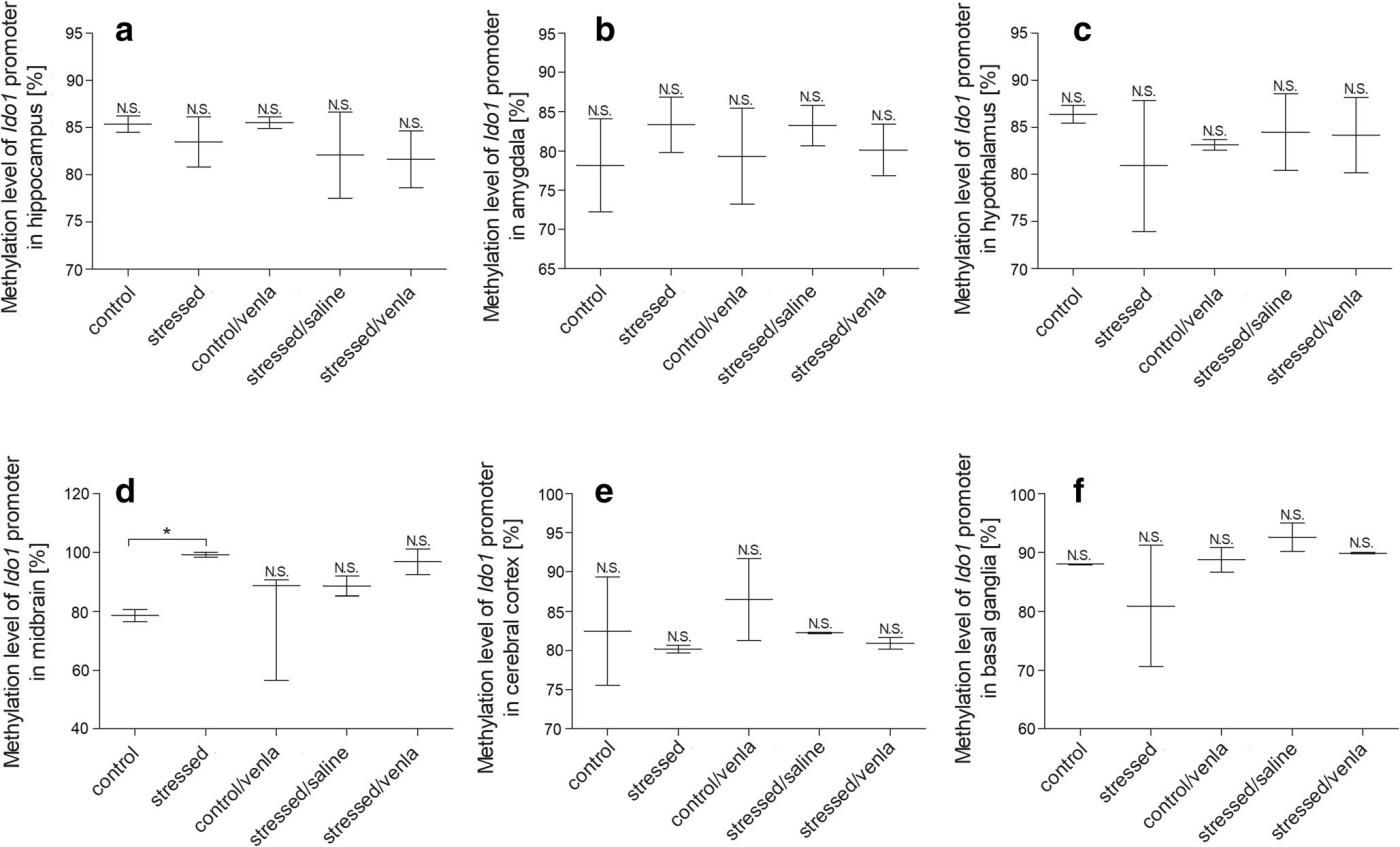
Methylation levels of *Ido1* promoter in hippocampus (**a**), amygdala (**b**), hypothalamus (**c**), midbrain (**d**), cerebral cortex (**e**) and basal ganglia (**f**) of animals exposed to CMS for 2 weeks (control, stressed) and in animals exposed to CMS for 7 weeks and treated with vehicle (1 ml/kg) or venlafaxine (10 mg/kg) for 5 weeks (control/venla, stressed/saline, stressed/venla). Data represent means ± SEM. *N* = 6. *p < 0.05 for differences between stressed and stressed/venla groups. *N.S.* no significant differences between studied groups

As shown in Fig. 3 and Supplementary Fig. 4, the methylation status of the *Tph1* (*p* < 0.001) and *Kmo* (*p* < 0.05) promoters in PBMCs was significantly increased in the venlafaxine-treated stressed group as compared with the stressed group, while the CMS procedure led to an elevated level of methylation of the *Ido1* promoter in the midbrain (Fig. 4 and Supplementary Table 4) (*p* < 0.05).

#### The Effect of Venlafaxine on the Methylation Status of Gene Promoters in PBMCs and Brain Structures

As shown in Supplementary Fig. 5, in stressed group venlafaxine decreased the methylation level of the *Tph1* promoter and *Kmo* promoter in all brain structures compared with the blood (*p* < 0.001, *p* < 0.01, respectively).

### Gene Expression at the Protein Level

**Fig. 5:**
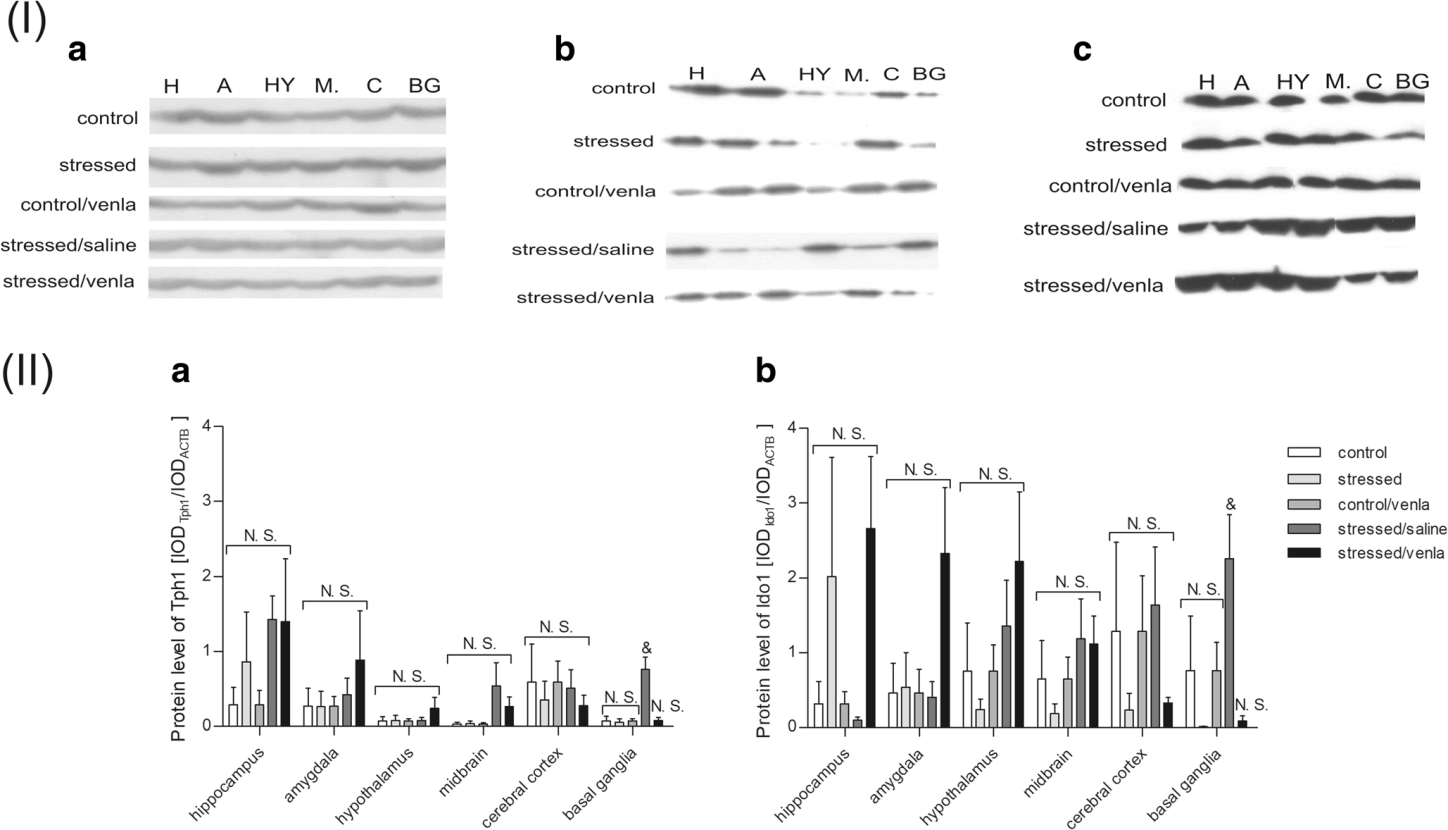
Protein expression of Tph1 (**a**) and Ido1 (**b**) in brain structures of animals exposed to CMS for 2 weeks (control, stressed) and in animals exposed to CMS for 7 weeks and administered vehicle (1 ml/kg) or venlafaxine (10 mg/kg) for 5 weeks (control/venla, stressed/saline, stressed/venla). (**I**) Representative western blot analysis in hippocampus (H), amygdala (A), hypothalamus (HY), midbrain (M), cerebral cortex (C) and basal ganglia (BG). A = β-actin, B = Tph1, C = Ido1. **(II)** Levels of Tph1 (**a**) and Ido1 (**b**) proteins measured in hippocampus, amygdala, hypothalamus, midbrain, cerebral cortex and basal ganglia. Samples containing 25 μg of proteins were resolved by SDS-PAGE. The intensity of bands corresponding to Gpx4 was analysed by densitometry. Integrated optical density (IOD) was normalized by protein content and a reference sample (see the Methods section for details). The graphs show the mean IODs of the bands from all analysed samples. The IOD_gene_/IOD_ACTB_ method was used to estimate the relative protein expression levels in the analysed samples. Data represent means ± SEM. *N* = 6. ^&^*p* < 0.05 for the difference between stressed/saline and stressed/venla groups. *N.S.* no significant differences between studied groups

The protein levels of Tph2, KatII and Kynu did not differ between the studied groups (Supplementary Fig. 6). Only the protein expression level of Tph1 (*p* < 0.05) and Ido1 (*p* < 0.05) (Fig. 5) was reduced in stressed animals administered venlafaxine as compared with the stressed group after saline treatment (*p* < 0.05).

## Discussion

The present study demonstrates the effect of the CMS model of depression and repeated administration of venlafaxine on the expression and methylation status of genes involved in the TRYCATs pathway in PBMCs and in six regions of the brain (hippocampus, amygdala, hypothalamus, midbrain, cerebral cortex and basal ganglia). In this study, genes expression were measured, rather than, activity of the antioxidant enzymes to highlight the role of epigenetic changes, i.e. promoter methylation, which is possible only when DNA, RNA and proteins are isolated from the same sample. All testes were done on tissue from animals after 5 weeks of CMS and venlafaxine, a point at which venlafaxine normalized behavioural impact of CMS.

Previous studies have shown that the CMS procedure leads to the development of depression-like behaviour, including anhedonia (Gamaro et al. 2003; Bekris et al. 2005, Papp 2012, Papp et al. 2017, 2019). The present study confirmed that CMS caused a reduction in the consumption of 1% sucrose solution, indicating a generalized deficit in sensitivity to reward, which is a characteristic symptom of depression, in stressed animals; this effect was normalized by the chronic administration of venlafaxine. Moreover, our results suggested that the CMS procedure and venlafaxine administration modulated the level of mRNA and protein expression and the status of the promoter methylation of genes that encode enzymes involved in the TRYCATs pathway in PBMCs and various brain structures, i.e., the hippocampus, amygdala, hypothalamus, midbrain, cerebral cortex and basal ganglia. Earlier animal studies confirmed that tryptophan insufficiency and serotonin disorders contribute to the development of depression-like symptoms (Tanke et al. 2008; Jacobsen et al. 2012). Likewise, clinical studies have also indicated that the TRYCATs pathway is dysfunctional in patients with depression (Myint 2012).

In our study, we focused on genes encoding enzymes involved in the TRYCATs pathway. The first of these is the gene encoding kynurenine aminotransferase (Kat). Kat catalyses the synthesis of kynurenic acid, which has neuroprotective properties and protects against neurodegenerative changes (Danzter 2017). Clinical studies have shown that the hippocampal and amygdala volume is reduced in depressed patients, which confirms the contribution of kynurenic acid generation imbalance to dendritic atrophy and anhedonia (Savitz et al. 2015). On the other hand, we found that the mRNA expression of *KatI* was lower in the hypothalamus and the cerebral cortex in the stressed animals administered venlafaxine, whereas stressed rats exhibited increased mRNA expression of *KatI* in the midbrain. We also found that the CMS procedure caused an elevated level of *KatII* mRNA expression in the amygdala and midbrain. However, Laugeray et al. (2010) observed that mice subjected to unpredictable CMS exhibit a reduced level of kynurenic acid in the amygdala and striatum. Similarly, CMS caused a downregulation of *KatII* mRNA expression in the cortex (Duda et al. 2019). These differences may be related to the fact that the analysed studies used a variety of biological material, including human, mouse and rat tissues. Additionally, the stress procedures used in the analysed studies were different. Thus, we cannot exclude the possibility that depression-like behaviour may be associated with the neurotoxic direction of kynurenine metabolism.

The next main finding of our study was that chronic venlafaxine administration in stressed rats decreased Tph1 protein expression in the basal ganglia and decreased the methylation status of the *Tph1* promoter region in PBMCs; however, no changes were observed in the brain. On the other hand, Chen et al. (2017) observed that stressed rats showed increased levels of *Tph1/Tph2* promoter methylation, which was normalized by paroxetine (an SSRI), in the brain, liver and kidney. These differences may be related to the fact that the stressors used in the above study were much more severe and that the animal breeds that were used differed between studies. Moreover, we found that the CMS procedure increased the mRNA expression of *Tph2* in the midbrain and that these effects were normalized by chronic venlafaxine administration. These findings are in line with clinical observations that an excess of *TPH2* may lead to tryptophan depletion and the development of depression-like symptoms (Jacobsen et al. 2012). On the other hand, TPH is involved in the initial and rate-limiting step of the synthesis of the neurotransmitter serotonin, and a low level of TPH may lead to the development of depression (McKinney et al. 2001; Cowen and Browning 2015). Thus, our results showed that antidepressant treatment in stressed rats, compared with placebo, caused an increase in the mRNA expression of *Tph2* in the cerebral cortex. Similarly, Jiao et al. (2019) showed that chronic immobilization stress (CIS) decreased the mRNA and protein expression of *Tph2* in the hippocampus, whereas fluoxetine normalized these effects.

The next studied gene encodes indoleamine 2,3-dioxygenase. A previous study confirmed that an increased level of *Ido1* leads to the overproduction of neurotoxic metabolites and a deficit in tryptophan. Additionally, high protein expression of *Ido1* may contribute to the inhibition of serotonin production (Myint et al. 2007; Wichers et al. 2005). Jiao et al. (2019) found that CIS increases the level of mRNA and protein expression in the hippocampus and that these effects are normalized by fluoxetine. In turn, our results showed that venlafaxine treatment in stressed rats caused a reduction in Ido1 protein expression in the basal ganglia. Moreover, citalopram therapy (an SSRI) also has the ability to inhibit the action of Ido1 as well as increase the turnover of serotonin via Ido1 inhibition in the hippocampus, amygdala and hypothalamus of stressed rats (Ara and Bano 2012). A similar effect associated with reduced depressive symptoms was observed in a group of animals administered Ido1 inhibitors and in *Ido1* knockout mice (O'Connor et al. 2009; Lawson et al. 2013; Liu et al. 2015, Salazar et al. 2012). These results may indicate the ability of antidepressant treatment to modulate the expression of enzymes involved in the TRYCATs pathway. Specifically, the studied drugs may limit the production of kynurenine and thus may reduce the levels of harmful metabolites and inhibit the neurodegenerative processes associated with the development of depression.

The next studied gene was *Kmo*. The only change we found was an increase in the methylation status of the *Kmo* promoter region in the PBMCs of stressed animals administered venlafaxine. Since the activity of the *Kmo* gene is associated with the production of neurotoxic 3-hydroxykynurenine and quinolinic acid and the generation of reactive oxygen species, the observed elevated methylation level might contribute to the reduction in Kmo activity and the impairment of neurons after venlafaxine administration (Guillemin 2012; Colin-Gonzalez et al. 2013). Moreover, the CMS procedure increased the mRNA expression level of *Kmo* in the cortex and may be associated with a higher level of the excitotoxic compound (Duda et al. 2019). However, Wang et al. (2018) found that rats subjected to the chronic unpredictable mild stress procedure exhibited diminished concentrations and reduced activity of *Kmo* in the plasma. These differences may be related to the fact that the stressors used in the above-mentioned study were much more severe and that the animal breeds used in the two studies were different. Additionally, all analysed studies were based on different biological materials, including PBMCs, cortex and plasma.

The present findings and those previously reported by others suggest that disorders of functioning enzymes involved in the TRYCATs pathway in PBMCs and the brain are involved in the effects observed in animals exposed to the CMS procedure. Antidepressant therapy, including venlafaxine, may modulate the activity of enzymes to restore tryptophan metabolism to its proper functioning. Moreover, our results and those of previous studies suggest that the stress stimuli and antidepressant therapy may modulate the methylation of promoter regions, with the effect that access of transcription factors to regulatory regions is reduced. However, depending on the type of regulatory agent, different changes are observed. If the regulatory binding site is for enhancers, DNA methylation is associated with transcriptional repression, whereas if the site is for repressors, DNA methylation will have the opposite effect on transcription (Murgatroyd et al. 2010; Jaenisch and Bird 2003). A traumatic and stressful event may cause epigenetic modification, including methylation, which may determine gene expression (Tsankova et al. 2007; Levenson and Sweatt 2005; Mill and Petronis 2007). We found that the CMS caused an increase in methylation status of the *Ido1* promoter. Previous studies have also confirmed that antidepressants, including tricyclic antidepressants (TCA) and SSRIs, may reduce DNA methylation in rat primary astrocytes by decreased activity of DNA methyltransferase 1 (Perisic et al. 2010; Zimmermann et al. 2012) Valproate, used as a mood stabilizer, may also cause a global reduction of DNA methylation level (Alonso-Aperte et al. 1999; Detich et al. 2003). Activation of DNA demethylation was also observed in the frontal cortex and striatum after treatment with the antipsychotics sulpiride and clozapine (Dong et al. 2008). On the other hand, we observed that venlafaxine therapy was associated with increased methylation levels of the *Tph1* and *Kmo* promoter regions. Interestingly, the DNA methylation level may determine the response to antidepressant treatment (Lopez et al. 2013; Domschke et al. 2014; Uher et al. 2009).

Epigenetic profiling may also be used for disease diagnosis and prognosis of disease progression and therapy effectiveness (Heyn and Esteller 2012). As previously noted, reduced gene expression is traditionally associated with elevated levels of methylation. However, the changes are dependent on binding of the enhancers or repressors to the affected sequence (Murgatroyd et al. 2010; Portela and Esteller 2010; Shukla et al. 2011; Mehta et al. 2013). The modification may be observed in DNA from the peripheral tissue (PBMCs) and brain (Provencal et al. 2012; Klengel et al. 2013; Suderman et al. 2012; Perroud et al. 2011). The epigenetic changes may be tissue-specific or may overlap. Thus, extrapolation of changes observed in one tissue to others should be made only with caution. Moreover, the epigenetic changes also include modification of histones and microRNAs (Mehler 2008). Therefore, we observed changes in expression levels without methylation modification.

## Conclusion

The results presented in this study confirm the hypothesis that the tryptophan catabolites pathway is involved in the pro-depressive effects of the CMS procedure. Moreover, disorders of tryptophan metabolites can be alleviated by venlafaxine. We also found that expression and methylation levels depend on the type of tissue (i.e., blood vs brain) as well as specific brain structure. In general, our results suggest that venlafaxine may prevent the overproduction of neurotoxic metabolites by inhibiting the protein expression of *Ido1* in the basal ganglia. We also confirmed that CMS causes a deficit in tryptophan through the mRNA expression of *Tph2* in the midbrain; however, this effect can be reversed by venlafaxine administration. Additionally, the hypermethylation of the *Kmo* promoter region may reduce the activity of the enzyme and limit the generation of neurotoxic 3-hydroxykynurenine and quinolinic acid.

## Electronic Supplementary Material

ESM 1 Supplementary Figure 1. mRNA expression *Tph1* (A) and *Kmo* (B) genes in brain structures of animals exposed to CMS for 2 weeks (control, stressed) and in animals exposed to CMS for 7 weeks and administered vehicle (1 ml/kg) or venlafaxine (10 mg/kg) for 5 weeks (control/venla, stressed/saline, stressed/venla). The expression of either gene was normalized to the 18S gene, and relative gene expression levels were estimated using a 2-ΔCt (Ct_gene_–Ct_18S_) method. *N* = 6; *N.S.* no significant differences between studied groups. (PNG 2760 kb)

High-resolution image (TIF 1225 kb)

ESM 2 Supplementary Figure 2. mRNA expression of *KatI* (A), *Kmo* (B) and *Kynu* (C) genes in PBMCs of animals exposed to CMS for 2 weeks (control, stressed) and in animals exposed to CMS for 7 weeks and administered vehicle (1 ml/kg) or venlafaxine (10 mg/kg) for 5 weeks (control/venla, stressed/saline, stressed/venla). Relative gene expression levels were estimated using a 2-ΔCt (Ctgene–Ct18S) method. Data represent means ± SEM. *N* = 6; *N.S.* no significant differences between studied groups. (PNG 2045 kb)

High-resolution image (TIF 1523 kb)

ESM 3 Supplementary Figure 3. mRNA expression of *Tph1* (A), *Tph2* (B), *KI* (C), *KATII* (D), *Kmo* (E) in PBMCs and in brain structures of animals exposed to CMS for 2 weeks (control, stressed) and in animals exposed to CMS for 7 weeks and administered vehicle (1 ml/kg) or venlafaxine (10 mg/kg) for 5 weeks (control/venla, stressed/saline, stressed/venla). The effects are presented as fold change (2^-ΔΔCt^ method; Schmittgen and Livak 2008). Data represent means ± SEM. *N* = 6. ***p < 0.001 and **p < 0.01 for differences between blood and all studied brain structures. (PNG 4883 kb)

High-resolution image (TIF 1914 kb)

ESM 4 Supplementary Figure 4. Methylation level of *Ido1* promoter (A), *Tdo2* promoter 1 (B) and *Tdo2* promoter 2 (C) in PBMCs of animals exposed to CMS for 2 weeks (control, stressed) and in animals exposed to CMS for 7 weeks and treated with vehicle (1 ml/kg) or venlafaxine (10 mg/kg) for 5 weeks (control/venla, stressed/saline, stressed/venla). Data represent means ± SEM. *N* = 6; *N.S.* no significant differences between studied groups. (PNG 2004 kb)

High-resolution image (TIF 415 kb)

ESM 5 Supplementary Figure 5. The methylation level of *Tph1* (A), *Ido1* (B), *Tdo2* promoter 1 (C), *Tdo2* promoter 2 (D) and *Kmo* (E) between brain structures and PBMCs of animals exposed to CMS for 2 weeks (control, stressed) and in animals exposed to CMS for 7 weeks and treated with vehicle (1 ml/kg) or venlafaxine (10 mg/kg) for 5 weeks (control/venla, stressed/saline, stressed/venla). Data represent means ± SEM. *N* = 6. *p < 0.05, **p < 0.01, ***p < 0.001 for differences between blood and all studied brain structures; *N.S.* no significant differences between studied groups. (PNG 4744 kb)

High-resolution image (TIF 940 kb)

ESM 6 Supplementary Figure 6. Expression of Tph2 (A), KatII(B) and Kynu (C) proteins in animals exposed to CMS for 2 weeks (control, stressed) and in animals exposed to CMS for 7 weeks and administered vehicle (1 ml/kg) or venlafaxine (10 mg/kg) for 5 weeks (control/venla, stressed/saline, stressed/venla). (I) Representative western blot analysis in hippocampus (H), amygdala (A), hypothalamus (HY), midbrain (M), cerebral cortex (C) and basal ganglia (BG). A = β-actin, B = Tph2, C = KatII, D = Kynu. (II) Levels of Tph2 (A), KatII (B) and Kynu (C) proteins measured in hippocampus, amygdala, hypothalamus, midbrain, cortex and basal ganglia. Samples containing 25 μg of proteins were resolved by SDS-PAGE. The intensity of the bands corresponding to Tph2, KATII and Kynu was analysed by densitometry, and integrated optical density (IOD) was normalized by protein content and a reference sample (see the Methods for details). The data show mean IODs of the bands from all analysed samples. The IOD_gene_/IOD_ACTB_ method was used to estimate the relative protein expression levels in the analysed samples. *N* = 6; *N.S.* no significant differences between studied groups. (PNG 5259 kb)

High-resolution image (TIF 28059 kb)

ESM 7 Supplementary Table 1. Characteristics of the genes studied (all data contained in the table were compiled with the help of the Genomatix Software Suite, Intrexon Bioinformatics Germany GmbH, Munich, Germany, 2019). (DOCX 14 kb)

ESM 8 Supplementary Table 2. The characteristics of primers used for analysis of methylation levels in the promoter regions of the studied genes. (DOCX 12 kb)

ESM 9 Supplementary Table 3. Conditions of the antibodies used in the Western blot analysis. (DOCX 12 kb)

ESM 10 Supplementary Table 4. Methylation level of *Ido1* promoter (A), *Tph1* promoter (B), *Tdo2* promoter 1 (C), *Tdo2* promoter 2 (D) and *Kmo* (E) in hippocampus, amygdala, hypothalamus, midbrain, cortex and basal ganglia of animals exposed to CMS for 2 weeks (control, stressed) and in animals exposed to CMS for 7 weeks and administered vehicle (1 ml/kg) or venlafaxine (10 mg/kg) for 5 weeks (control/venla, stressed/saline, stressed/venla). Data represent means ± SEM. *N* = 6. No significant changes were found between any groups. (DOCX 16 kb)
