# Supplementary figures and images for: The Effect of Chronic Mild Stress and Venlafaxine on the Expression and Methylation Levels of Genes Involved in the Tryptophan Catabolites Pathway in the Blood and Brain Structures of Rats
Source: J Mol Neurosci. 2020 May 13;70(9):1425–36. doi: 10.1007/s12031-020-01563-2 (PMC7399689; doi:10.1007/s12031-020-01563-2)

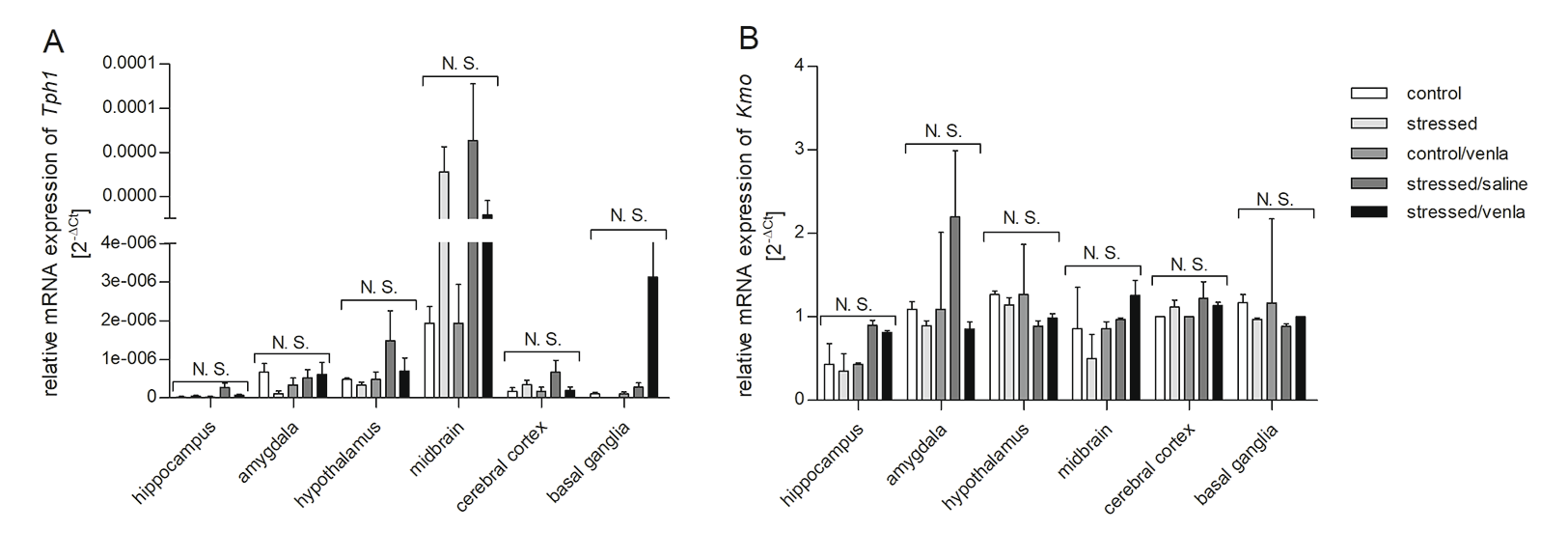

Supplement: Supplementary file 1 — Supplementary Figure 1. mRNA expression Tph1 (A) and Kmo (B) genes in brain structures of animals exposed to CMS for 2 weeks (control, stressed) and in animals exposed to CMS for 7 weeks and administered vehicle (1 ml/kg) or venlafaxine (10 mg/kg) for 5 weeks (control/venla, stressed/saline, stressed/venla). The expression of either gene was normalized to the 18S gene, and relative gene expression levels were estimated using a 2-ΔCt (Ctgene–Ct18S) method. N = 6; N.S. no significant differences between studied groups. (PNG 2760 kb) [file 12031_2020_1563_Fig6_ESM.png]

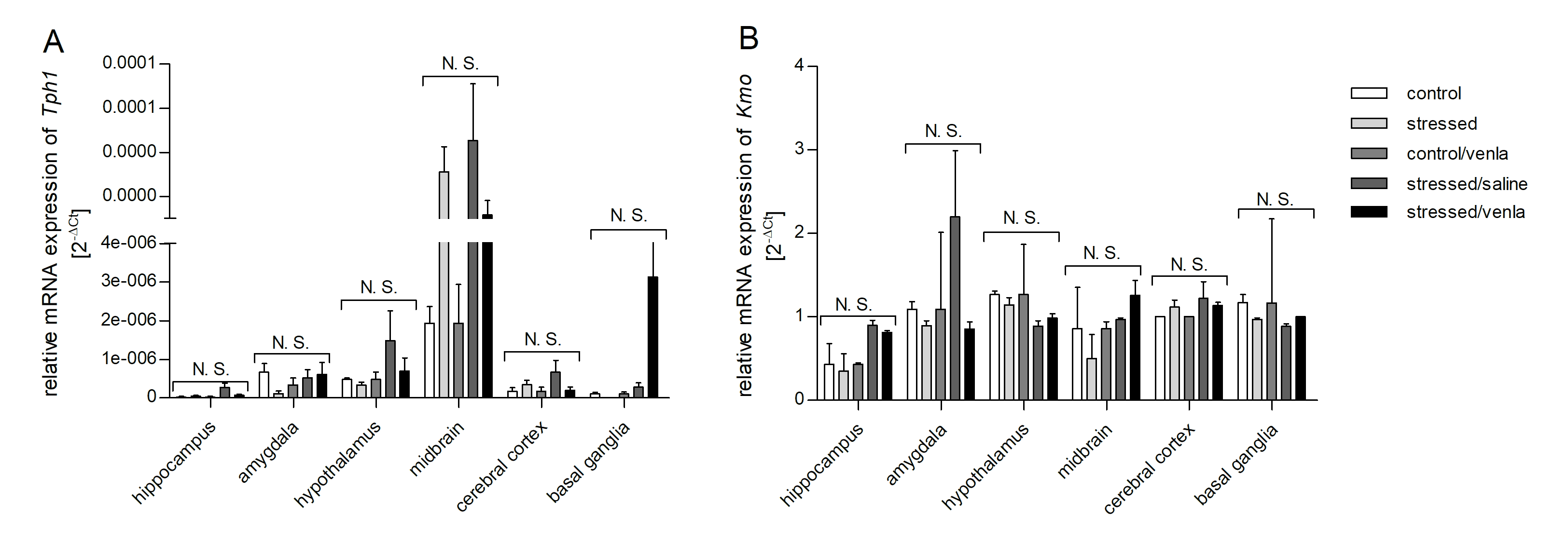

Supplement: Supplementary file 2 — High-resolution image (TIF 1225 kb) [file 12031_2020_1563_MOESM1_ESM.tif]

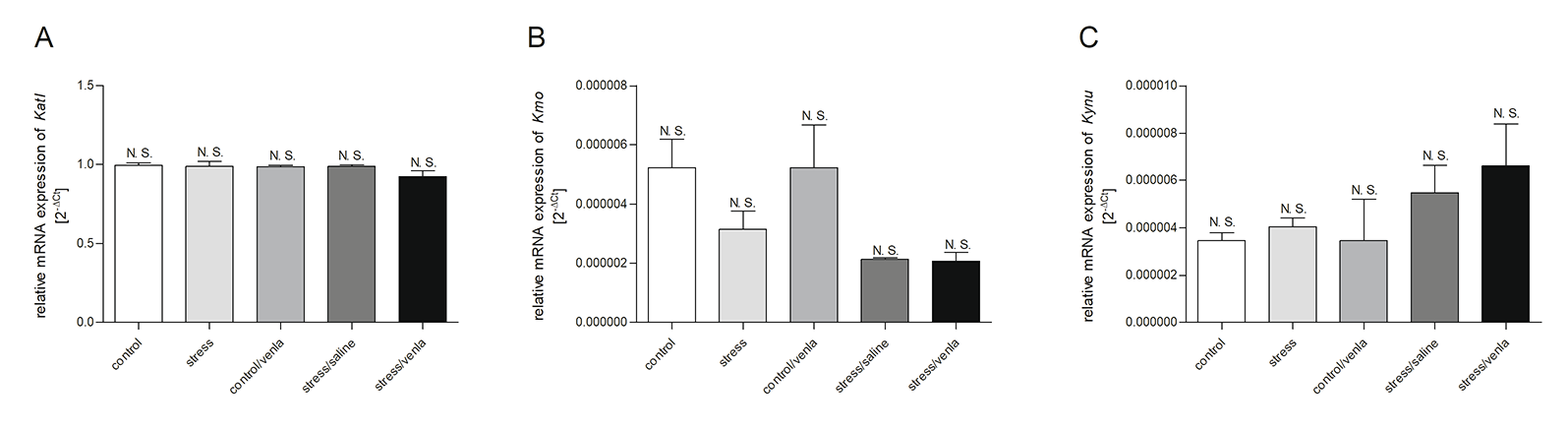

Supplement: Supplementary file 3 — Supplementary Figure 2. mRNA expression of KatI (A), Kmo (B) and Kynu (C) genes in PBMCs of animals exposed to CMS for 2 weeks (control, stressed) and in animals exposed to CMS for 7 weeks and administered vehicle (1 ml/kg) or venlafaxine (10 mg/kg) for 5 weeks (control/venla, stressed/saline, stressed/venla). Relative gene expression levels were estimated using a 2-ΔCt (Ctgene–Ct18S) method. Data represent means ± SEM. N = 6; N.S. no significant differences between studied groups. (PNG 2045 kb) [file 12031_2020_1563_Fig7_ESM.png]

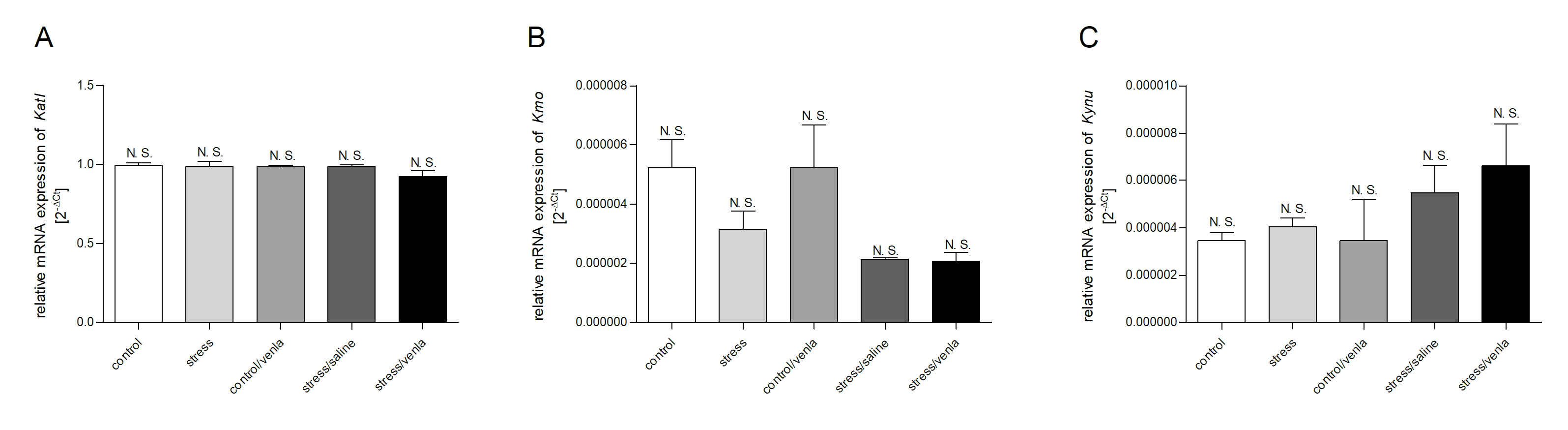

Supplement: Supplementary file 4 — High-resolution image (TIF 1523 kb) [file 12031_2020_1563_MOESM2_ESM.tif]

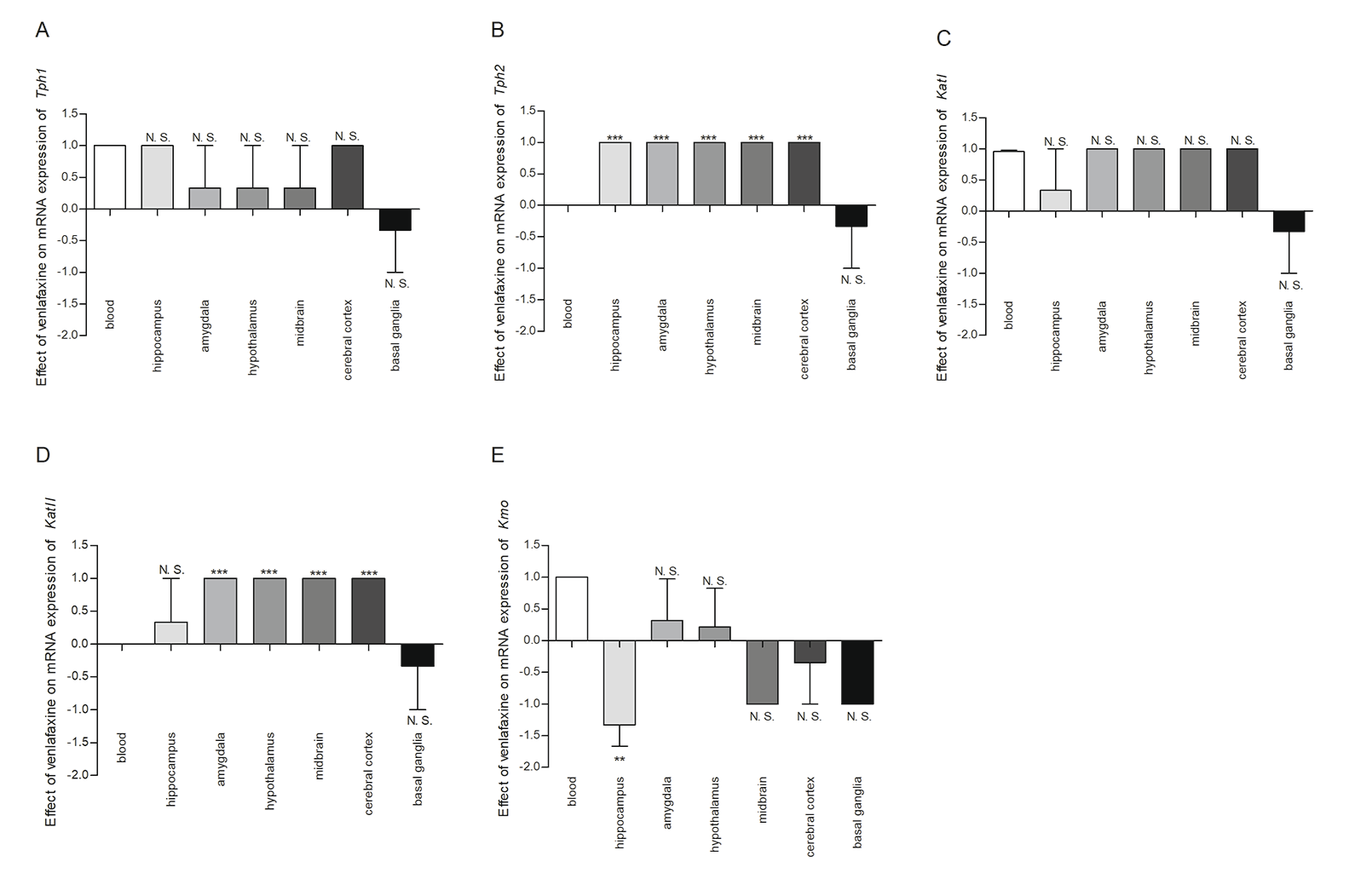

Supplement: Supplementary file 5 — Supplementary Figure 3. mRNA expression of Tph1 (A), Tph2 (B), KI (C), KATII (D), Kmo (E) in PBMCs and in brain structures of animals exposed to CMS for 2 weeks (control, stressed) and in animals exposed to CMS for 7 weeks and administered vehicle (1 ml/kg) or venlafaxine (10 mg/kg) for 5 weeks (control/venla, stressed/saline, stressed/venla). The effects are presented as fold change (2-ΔΔCt method; Schmittgen and Livak 2008). Data represent means ± SEM. N = 6. ***p < 0.001 and **p < 0.01 for differences between blood and all studied brain structures. (PNG 4883 kb) [file 12031_2020_1563_Fig8_ESM.png]

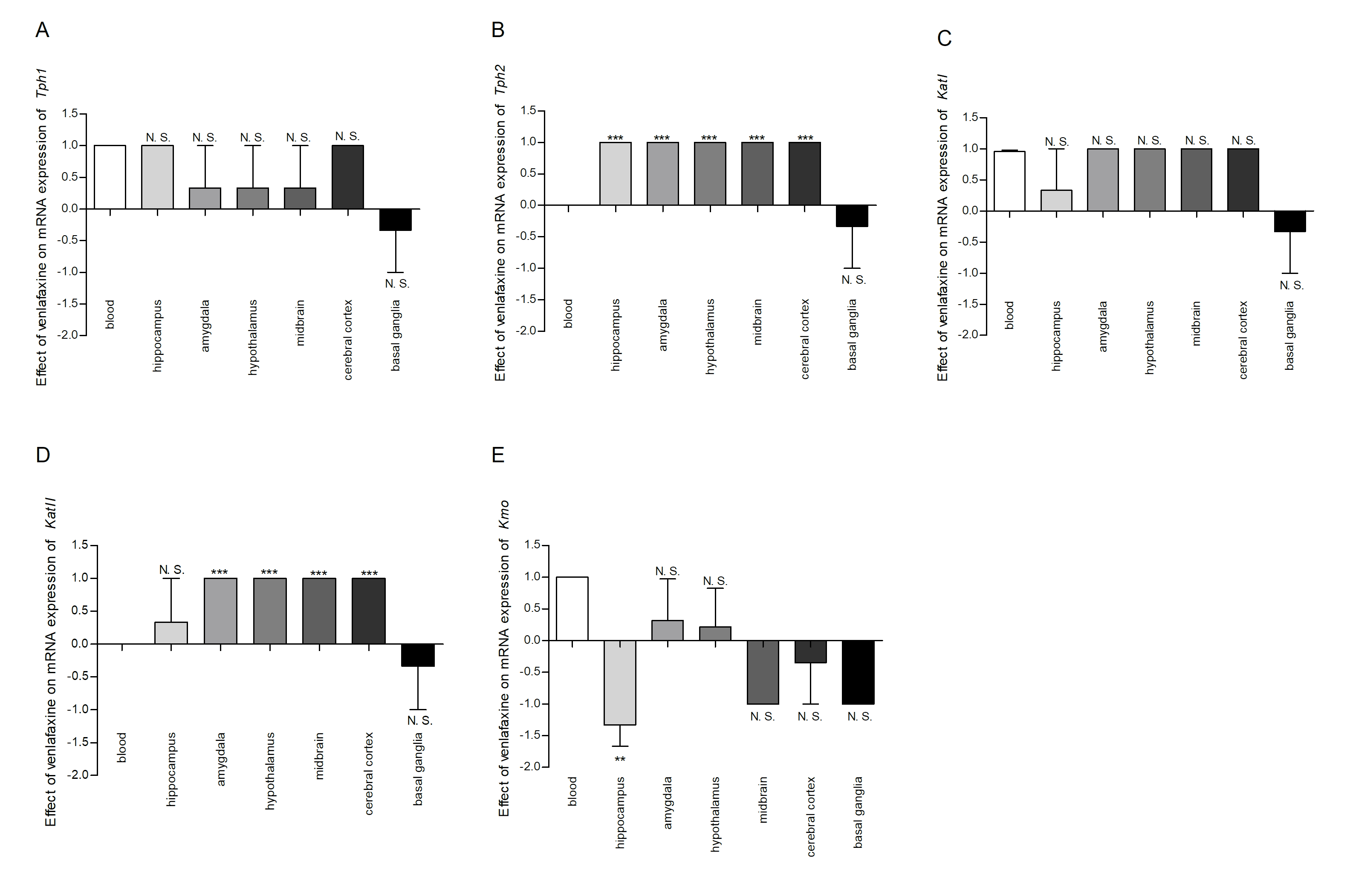

Supplement: Supplementary file 6 — High-resolution image (TIF 1914 kb) [file 12031_2020_1563_MOESM3_ESM.tif]

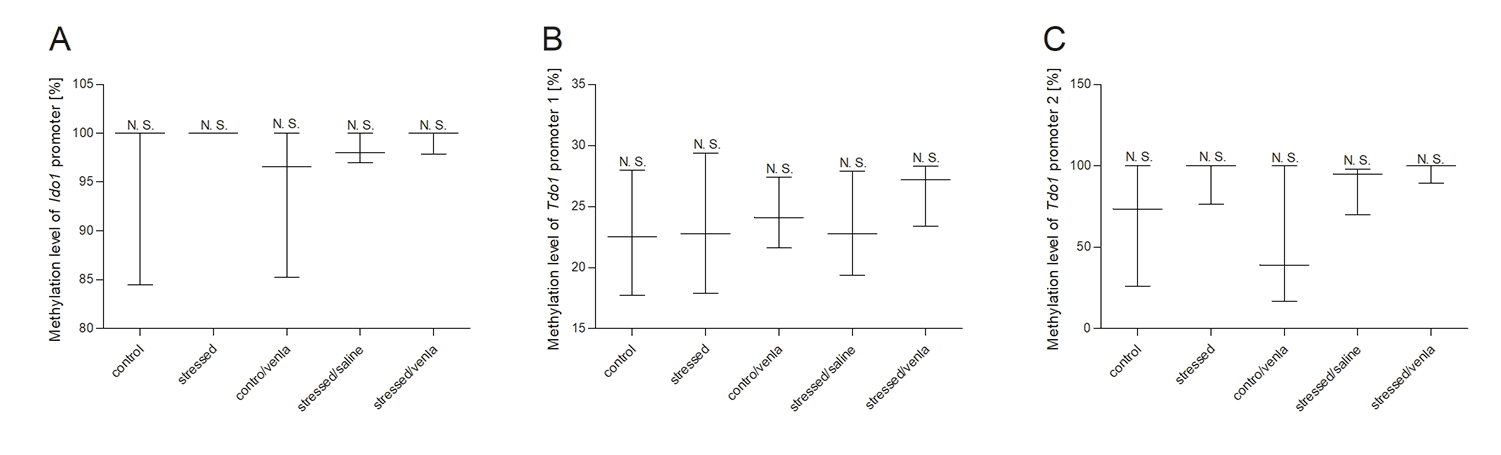

Supplement: Supplementary file 7 — Supplementary Figure 4. Methylation level of Ido1 promoter (A), Tdo2 promoter 1 (B) and Tdo2 promoter 2 (C) in PBMCs of animals exposed to CMS for 2 weeks (control, stressed) and in animals exposed to CMS for 7 weeks and treated with vehicle (1 ml/kg) or venlafaxine (10 mg/kg) for 5 weeks (control/venla, stressed/saline, stressed/venla). Data represent means ± SEM. N = 6; N.S. no significant differences between studied groups. (PNG 2004 kb) [file 12031_2020_1563_Fig9_ESM.png]

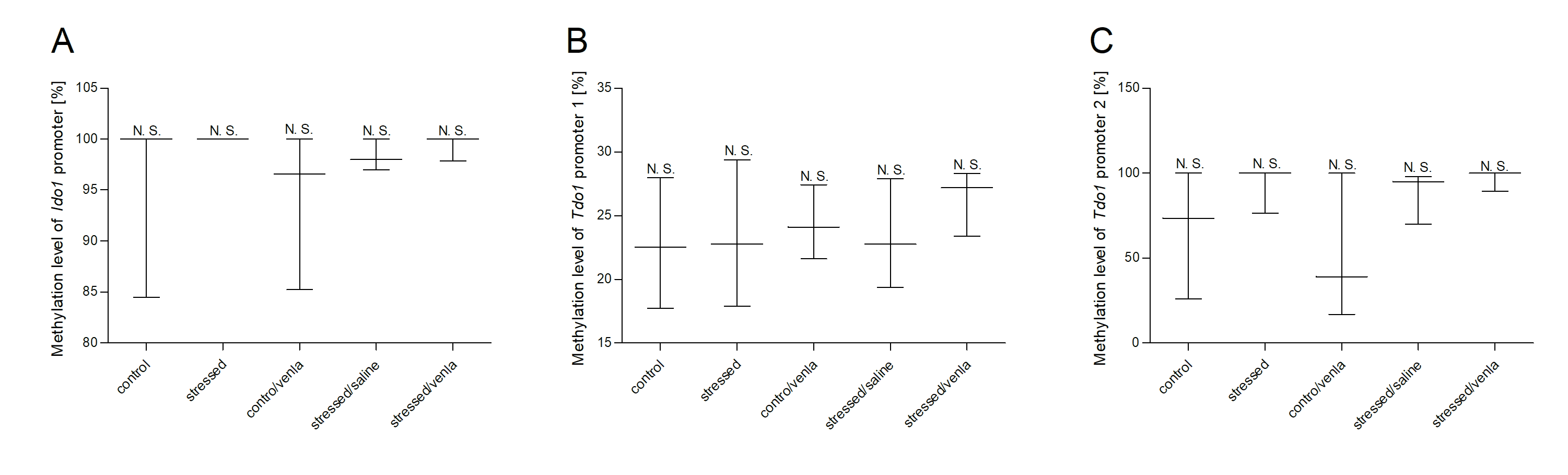

Supplement: Supplementary file 8 — High-resolution image (TIF 415 kb) [file 12031_2020_1563_MOESM4_ESM.tif]

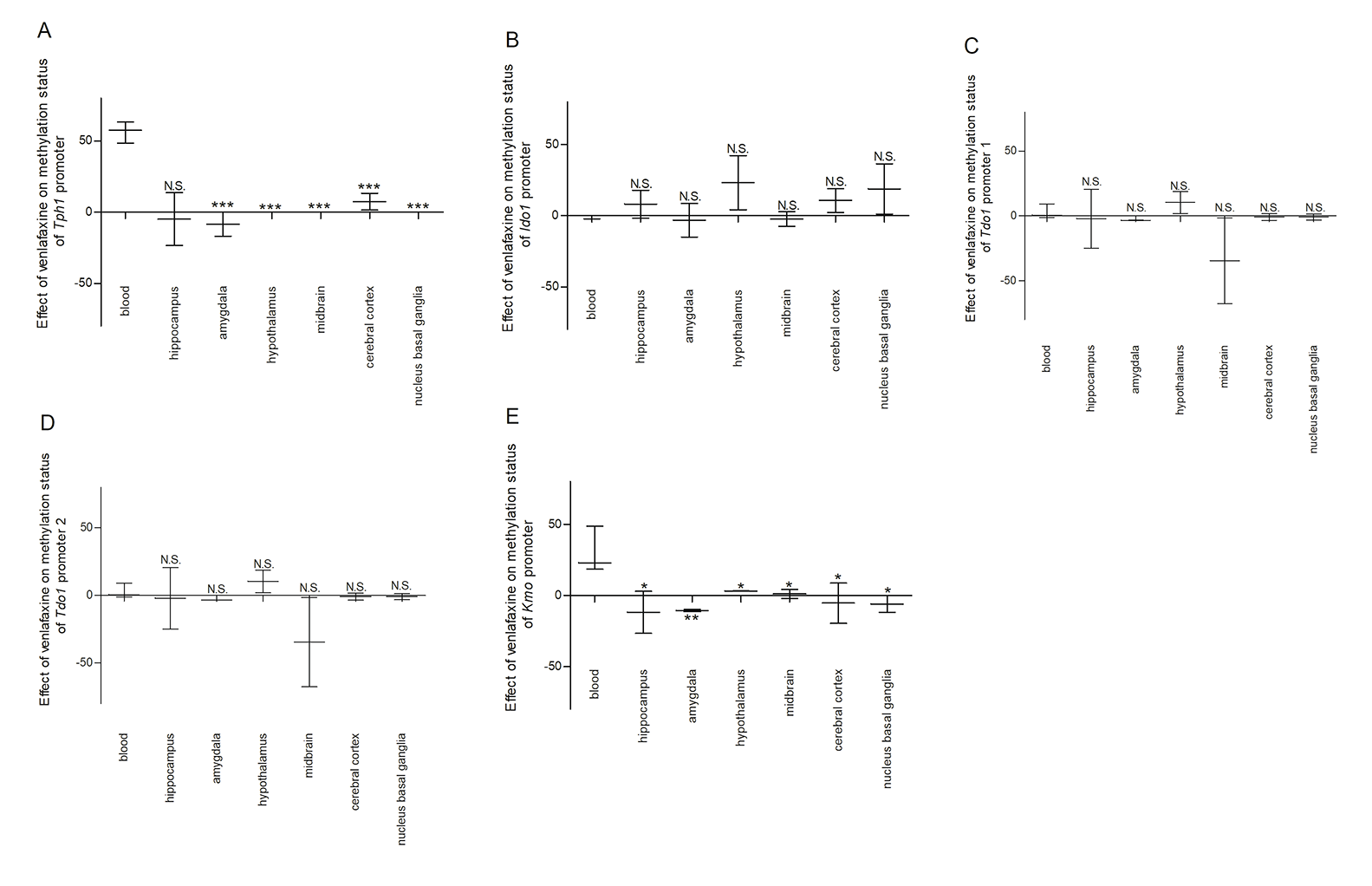

Supplement: Supplementary file 9 — Supplementary Figure 5. The methylation level of Tph1 (A), Ido1 (B), Tdo2 promoter 1 (C), Tdo2 promoter 2 (D) and Kmo (E) between brain structures and PBMCs of animals exposed to CMS for 2 weeks (control, stressed) and in animals exposed to CMS for 7 weeks and treated with vehicle (1 ml/kg) or venlafaxine (10 mg/kg) for 5 weeks (control/venla, stressed/saline, stressed/venla). Data represent means ± SEM. N = 6. *p < 0.05, **p < 0.01, ***p < 0.001 for differences between blood and all studied brain structures; N.S. no significant differences between studied groups. (PNG 4744 kb) [file 12031_2020_1563_Fig10_ESM.png]

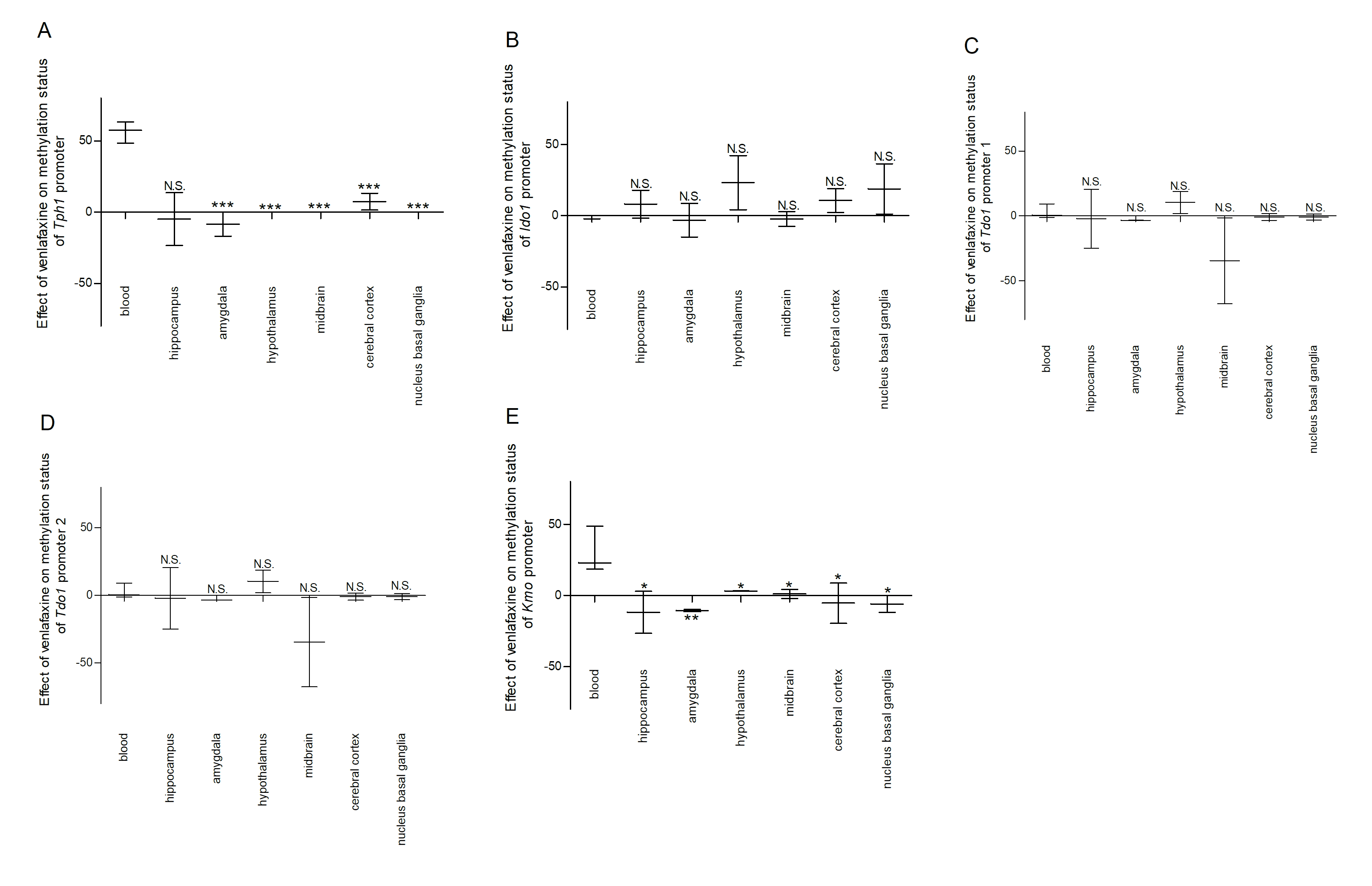

Supplement: Supplementary file 10 — High-resolution image (TIF 940 kb) [file 12031_2020_1563_MOESM5_ESM.tif]

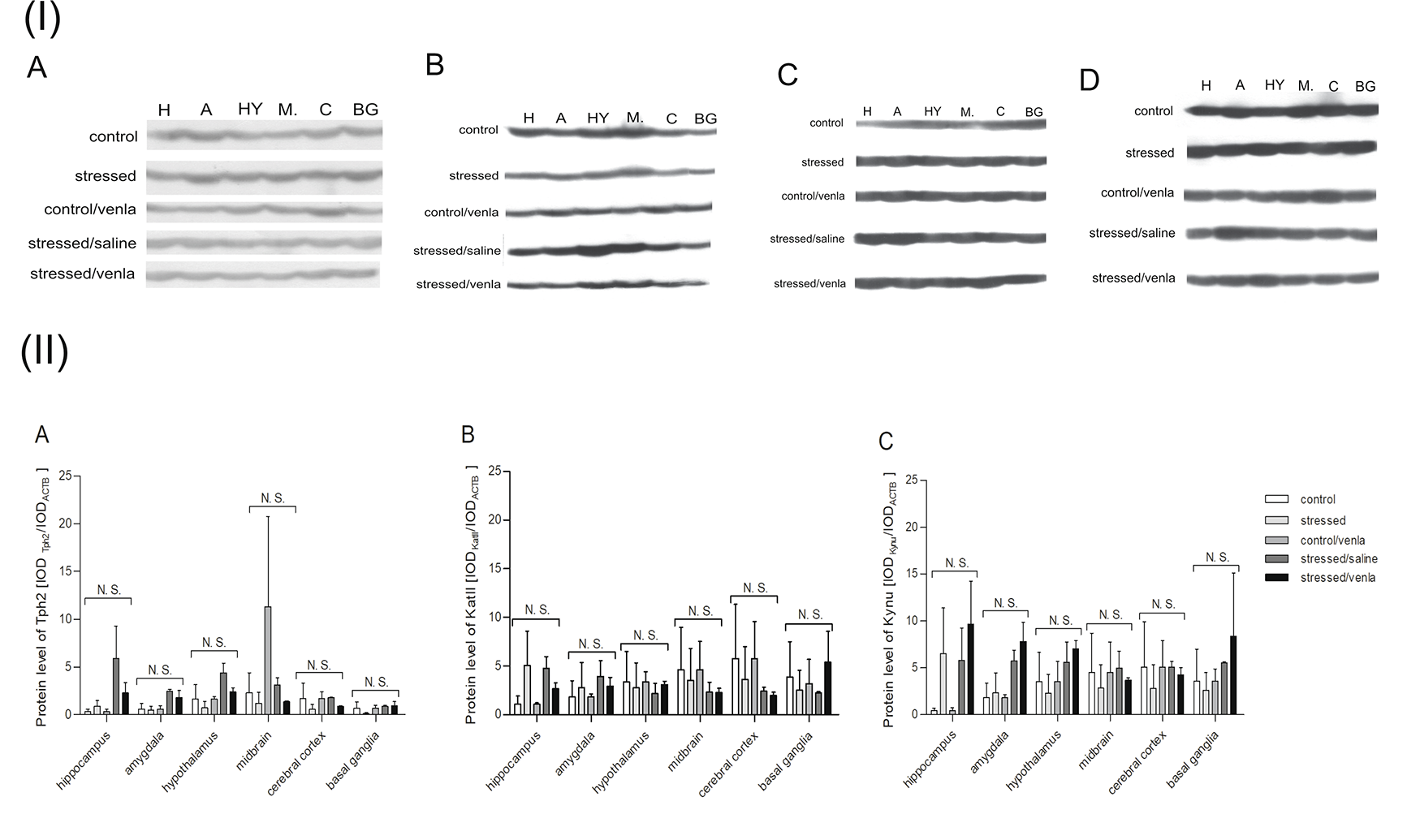

Supplement: Supplementary file 11 — Supplementary Figure 6. Expression of Tph2 (A), KatII(B) and Kynu (C) proteins in animals exposed to CMS for 2 weeks (control, stressed) and in animals exposed to CMS for 7 weeks and administered vehicle (1 ml/kg) or venlafaxine (10 mg/kg) for 5 weeks (control/venla, stressed/saline, stressed/venla). (I) Representative western blot analysis in hippocampus (H), amygdala (A), hypothalamus (HY), midbrain (M), cerebral cortex (C) and basal ganglia (BG). A = β-actin, B = Tph2, C = KatII, D = Kynu. (II) Levels of Tph2 (A), KatII (B) and Kynu (C) proteins measured in hippocampus, amygdala, hypothalamus, midbrain, cortex and basal ganglia. Samples containing 25 μg of proteins were resolved by SDS-PAGE. The intensity of the bands corresponding to Tph2, KATII and Kynu was analysed by densitometry, and integrated optical density (IOD) was normalized by protein content and a reference sample (see the Methods for details). The data show mean IODs of the bands from all analysed samples. The IODgene/IODACTB method was used to estimate the relative protein expression levels in the analysed samples. N = 6; N.S. no significant differences between studied groups. (PNG 5259 kb) [file 12031_2020_1563_Fig11_ESM.png]

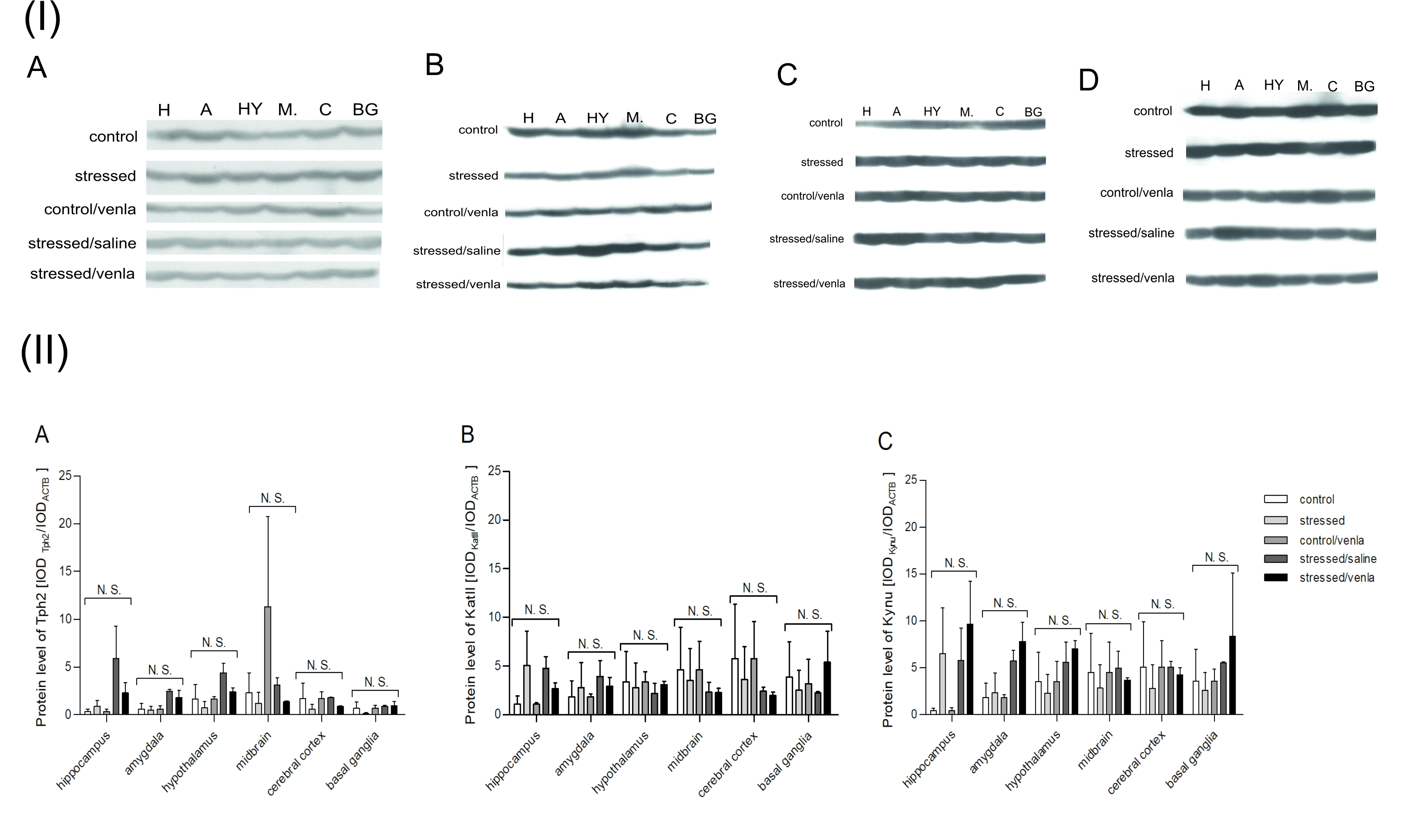

Supplement: Supplementary file 12 — High-resolution image (TIF 28059 kb) [file 12031_2020_1563_MOESM6_ESM.tif]
