# Supplementary material for: The Effect of Chronic Mild Stress and Venlafaxine on the Expression and Methylation Levels of Genes Involved in the Tryptophan Catabolites Pathway in the Blood and Brain Structures of Rats
Source: J Mol Neurosci. 2020 May 13;70(9):1425–36. doi: 10.1007/s12031-020-01563-2 (PMC7399689; doi:10.1007/s12031-020-01563-2)
Supplement: Supplementary file 13 — Supplementary Table 1. Characteristics of the genes studied (all data contained in the table were compiled with the help of the Genomatix Software Suite, Intrexon Bioinformatics Germany GmbH, Munich, Germany, 2019). (DOCX 14 kb) [file 12031_2020_1563_MOESM7_ESM.docx]

**Supplementary Table 1.** Characteristics of the genes studied (All data contained in the table were compiled with the help of Genomatix Software Suite, Intrexon Bioinformatics Germany GmbH, Munich, Germany, 2019).

| Gene name | Function of protein  encoding the gene | Chromosomal location | Biased expression |
| --- | --- | --- | --- |
| Cysteine conjugate-beta lyase 1 (*KatI*) | Catalyzes the irreversible transamination of the L-tryptophan metabolite L-Kynurenine to form Kynurenic acid | 3p12 | Adrenal gland, brain, heart, kidney, liver, lung, muscle, spleen, thymus, testes, uterus |
| 2-aminoadipate aminotransferase (*KatII*) | Endogenous modulator of glutamatergic neurotransmission with Kynurenine aminotransferase (KAT) activity | 16p12 | Kidney,  liver |
| Tryptophan hydroxylase 1 (*Tph1*) | Catalyzes the conversion of l-tryptophanof to 5-hydroxy-l-tryptophan, member of pterin-dependent aromatic amino acid hydroxylase family | 1q22 | Thymus, brain, kidney, muscle, testes |
| Tryptophan hydroxylase 2 (*Tph2*) | Isoform of tryptophan hydroxylase, may play a role in serotonin biosynthesis | 7q22 | Adrenal, brain, testes |
| Indoleamine-2,3-dioxygenase (Ido1) | Enzyme which catalyses degradation of L-tryptophan to N-formylkynurenine; human homolog is induced by gamma-interferon and is belived to be involved in that agent’s tumor antiproliferative effects | 16q12.5 | Testes, thymus, heart, lung |
| Tryptophan 2,3-dioxygenase (*Tdo2*) | Rate-limiting in the catabolism of tryptophan | 2q34 | Liver, lung |
| Kynurenine 3-hydroxylase (*Kmo*) | Catalyses the hydroxylation of L-Kynurenine to form L-3-hydroxyKynurenine | 13q25 | Kidney, liver, lung, spleen, thymus, uterus |
| Kynureninase (*Kynu*) | Enzyme involved in the biosynthesis of NAD cofactors from tryptophan | 3q12 | Kidney, liver, spleen |
