# Supplementary material for: The Effect of Chronic Mild Stress and Venlafaxine on the Expression and Methylation Levels of Genes Involved in the Tryptophan Catabolites Pathway in the Blood and Brain Structures of Rats
Source: J Mol Neurosci. 2020 May 13;70(9):1425–36. doi: 10.1007/s12031-020-01563-2 (PMC7399689; doi:10.1007/s12031-020-01563-2)
Supplement: Supplementary file 14 — Supplementary Table 2. The characteristics of primers used for analysis of methylation levels in the promoter regions of the studied genes. (DOCX 12 kb) [file 12031_2020_1563_MOESM8_ESM.docx]

**Supplementary table 2.** The characteristics of primers used for analysis of methylation levels in the promoter regions of the studied genes.

| **Gene** | **Starter sequence** | **Product size** | **Tm** |
| --- | --- | --- | --- |
| ***Tph1 (promoter 2)*** | F:GGGAGTTTTGTTTTGGTTTTTA  R:TCCTCAACCACAAAAAATCTAA | 132 | 55 |
| ***Ido1 (promoter 2)*** | F:TTTGAGTTTTAGTGATTTTGGG  R:TTAATATCTAATCCCAATCTCTAAAAC | 100 | 59 |
| ***Tdo2 (promoter 1)*** | F:GATGATTTAGGTGGTTTGAGGT  R:CAAAAAAAACAAAATTCATCCA | 123 | 59 |
| ***TDO2 (promoter 2)*** | F:ATGATTTAGGTGGTTTGAGGTT  R:ACCCAATCTACCTAACTAACAAC | 187 | 61.4 |
| ***Kmo (promoter 7)*** | F: TTGGTTTAGGGAAGGAAAT  R: ATAAAAAACTAAACCCAAAACAC | 150 | 55.7 |
