# Supplementary material for: The Effect of Chronic Mild Stress and Venlafaxine on the Expression and Methylation Levels of Genes Involved in the Tryptophan Catabolites Pathway in the Blood and Brain Structures of Rats
Source: J Mol Neurosci. 2020 May 13;70(9):1425–36. doi: 10.1007/s12031-020-01563-2 (PMC7399689; doi:10.1007/s12031-020-01563-2)
Supplement: Supplementary file 15 — Supplementary Table 3. Conditions of the antibodies used in the Western blot analysis. (DOCX 12 kb) [file 12031_2020_1563_MOESM9_ESM.docx]

**Supplementary table 3.** Conditions of the antibodies used in the Western blot analysis.

|  | **Primary antibody** | **Secondary antibody** |
| --- | --- | --- |
| **β-actin**  **(a reference protein)** | mouse, 1:1000, (Santa Cruz Biotechnolgy Inc),  1 hour at room temperature | anti-mouse, 1:6000, (Cell Signalling Technologies Inc., Danvers, Massachusetts, USA),  1 hour at room temperature |
| **Tryptophan hydroxylase 1** | rabbit, 1:1000, (Cell Signalling Technologies Inc., Danvers, Massachusetts, USA),  overnight at 4°C | anti-rabbit, 1:6000, (Cell Signalling Technologies Inc., Danvers, Massachusetts, USA),  1 hour at room temperature |
| **Tryptophan hydroxylase 2** | rabbit, 1:6000, (Cell Signalling Technologies Inc., Danvers, Massachusetts, USA),  overnight at 4°C | anti-rabbit, 1:6000, (Cell Signalling Technologies Inc., Danvers, Massachusetts, USA),  1 hour at room temperature |
| **Indoleamine 2,3-dioxygenase** | mouse, 1:1000, (Santa Cruz Biotechnolgy Inc), overnight at 4°C | anti-mouse, 1:6000, (Cell Signalling Technologies Inc., Danvers, Massachusetts, USA),  1 hour at room temperature |
| **Kynurenine aminotransferases** | mouse, 1:1000, (Santa Cruz Biotechnolgy Inc), overnight at 4°C | anti-mouse, 1:6000, (Cell Signalling Technologies Inc., Danvers, Massachusetts, USA),  1 hour at room temperature |
| **Kynureninase** | mouse, 1:1000, (Santa Cruz Biotechnolgy Inc), overnight at 4°C | anti-mouse, 1:6000, (Cell Signalling Technologies Inc., Danvers, Massachusetts, USA),  1 hour at room temperature |
