# Supplementary material for: The Effect of Chronic Mild Stress and Venlafaxine on the Expression and Methylation Levels of Genes Involved in the Tryptophan Catabolites Pathway in the Blood and Brain Structures of Rats
Source: J Mol Neurosci. 2020 May 13;70(9):1425–36. doi: 10.1007/s12031-020-01563-2 (PMC7399689; doi:10.1007/s12031-020-01563-2)
Supplement: Supplementary file 16 — Supplementary Table 4. Methylation level of Ido1 promoter (A), Tph1 promoter (B), Tdo2 promoter 1 (C), Tdo2 promoter 2 (D) and Kmo (E) in hippocampus, amygdala, hypothalamus, midbrain, cortex and basal ganglia of animals exposed to CMS for 2 weeks (control, stressed) and in animals exposed to CMS for 7 weeks and administered vehicle (1 ml/kg) or venlafaxine (10 mg/kg) for 5 weeks (control/venla, stressed/saline, stressed/venla). Data represent means ± SEM. N = 6. No significant changes were found between any groups. (DOCX 16 kb) [file 12031_2020_1563_MOESM10_ESM.docx]

**Supplementary table 4**. Methylation level of, *Tph1* promoter (A), *TDO2* promoter 1 (B), *TDO2* promoter 2 (C) and *Kmo* (D) in hippocampus, amygdala, hypothalamus, midbrain, cortex and basal ganglia of animals exposed to CMS for two weeks (control, stressed) and in animals exposed to CMS for seven weeks and administered vehicle (1 ml/kg) or venlafaxine (10 mg/kg) for five weeks (control/venla, stressed/saline, stressed/venla). Data represents means ± SEM. N = 6. No significant changes were found between any groups.

(A)

| Methylation level of *Tph1* promoter | | | | | |
| --- | --- | --- | --- | --- | --- |
| Part of brain | Control | Stressed | Control/Venla | Stressed/Saline | Stressed/Venla |
| Hippocampus | 95.90 ± 2.37 | 93.11 ± 3.98 | 96.62 ± 3.17 | 99.60 ± 0.20 | 88.29 ± 6.76 |
| Amygdala | 98.76 ± 0.71 | 99.15 ± 0.54 | 99.73 ± 0.12 | 100.00 ± 0.00 | 91.54 ± 4.88 |
| Hypothalamus | 98.01 ± 0.51 | 99.23 ± 0.43 | 99.01 ± 0.66 | 96.07 ± 2.27 | 99.76 ± 0.11 |
| Midbrain | 91.34 ± 0.38 | 98.54 ± 0.43 | 90.99 ± 0.21 | 99.65 ± 0.21 | 99.65 ± 0.31 |
| Cerebral cortex | 99.01 ± 0.31 | 92.57 ± 3.34 | 99.52 ± 0.41 | 96.09 ± 2.26 | 98.54 ± 0.15 |
| Basal ganglia | 98.65 ± 0.76 | 99.54 ± 0.11 | 97.21 ± 0.77 | 99.54 ± 0.15 | 99.01 ± 0.75 |

(B)

| \| Methylation level of *TDO2* promoter 1 \| \| \| \| \| \| \| --- \| --- \| --- \| --- \| --- \| --- \| \| Part of brain \| Control \| Stressed \| Control/Venla \| Stressed/Saline \| Stressed/Venla \| \| Hippocampus \| 61.99 ± 21.95 \| 12.45 ± 7.19 \| 55.66 ± 16.53 \| 23.58 ± 0.05 \| 10.36 ± 5.98 \| \| Amygdala \| 9.21 ± 8.21 \| 25.02 ± 0.74 \| 11.16 ± 5.98 \| 24.10 ± 0.21 \| 21.61 ± 0.81 \| \| Hypothalamus \| 11.65 ± 6.73 \| 12.14 ± 7.01 \| 8.87 ± 6.87 \| 12.19 ± 7.04 \| 22.55 ± 2.09 \| \| Midbrain \| 24.12 ± 0.50 \| 58.12 ± 17.62 \| 27.76 ± 6.75 \| 25.10 ± 0.26 \| 23.57 ± 1.50 \| \| Cerebral cortex \| 12.12 ± 7.00 \| 24.28 ± 0.41 \| 21.77 ± 9.21 \| 0.00 ± 0.00 \| 23.45 ± 1.10 \| \| Basal ganglia \| 25.70 ± 0.45 \| 24.52 ± 0.15 \| 20.12 ± 2.54 \| 24.83 ± 0.02 \| 23.66 ± 1.49 \| |
| --- | --- | --- | --- | --- | --- | --- | --- | --- | --- | --- | --- | --- | --- | --- | --- | --- | --- | --- | --- | --- | --- | --- | --- | --- | --- | --- | --- | --- | --- | --- | --- | --- | --- | --- | --- | --- | --- | --- | --- | --- | --- | --- | --- | --- | --- | --- | --- | --- |

(C)

| Methylation level of *TDO2* promoter 2 | | | | | |
| --- | --- | --- | --- | --- | --- |
| Part of brain | Control | Stressed | Control/Venla | Stressed/Saline | Stressed/Venla |
| Hippocampus | 0.00 ± 0.00 | 12.14 ± 7.01 | 0.00 ± 0.00 | 0.00 ± 0.00 | 9.84 ± 5.68 |
| Amygdala | 0.00 ± 0.00 | 0.00 ± 0.00 | 0.00 ± 0.00 | 0.00 ± 0.00 | 0.00 ± 0.00 |
| Hypothalamus | 0.00 ± 0.00 | 0.00 ± 0.00 | 0.00 ± 0.00 | 0.00 ± 0.00 | 13.11 ± 7.57 |
| Midbrain | 0.00 ± 0.00 | 16.33 ± 9.43 | 0.00 ± 0.00 | 0.00 ± 0.00 | 30.96 ± 2.74 |
| Cerebral cortex | 0.00 ± 0.00 | 11.56 ± 6.67 | 0.00 ± 0.00 | 0.00 ± 0.00 | 2.26 ± 1.30 |
| Basal ganglia | 0.93 ± 0.54 | 0.00 ± 0.00 | 0.83 ± 0.45 | 0.00 ± 0.00 | 29.84 ± 17.23 |

(D)

| Methylation level of *Kmo* promoter | | | | | |
| --- | --- | --- | --- | --- | --- |
| Part of brain | Control | Stressed | Control/Venla | Stressed/Saline | Stressed/Venla |
| Hippocampus | 96.43 ± 1.81 | 88.65 ± 2.96 | 94.66 ± 1.43 | 89.49 ± 3.27 | 76.83 ± 5.60 |
| Amygdala | 98.40 ± 0.92 | 96.75 ± 1.88 | 96.51 ± 1.27 | 97.07 ± 7.89 | 86.31 ± 2.35 |
| Hypothalamus | 94.32 ± 0.96 | 88.66 ± 3.57 | 96.76 ± 1.05 | 86.76 ± 0.28 | 91.85 ± 3.54 |
| Midbrain | 97.10 ± 1.67 | 96.77 ± 1.87 | 93.09 ± 3.87 | 97.79 ± 1.27 | 98.00 ± 0.01 |
| Cerebral cortex | 91.50 ± 3.20 | 75.10 ± 5.11 | 94.65 ± 4.76 | 67.24 ± 10.19 | 69.82 ± 13.31 |
| Basal ganglia | 100.00 ± 0.00 | 99.76 ± 0.11 | 99.53 ± 0.32 | 99.91 ± 0.05 | 94.08 ± 3.42 |
